# Long-Term High-Fat High-Fructose Diet Induces Type 2 Diabetes in Rats through Oxidative Stress

**DOI:** 10.3390/nu14112181

**Published:** 2022-05-24

**Authors:** Yue Zhao, Qing-Yu Wang, Lv-Tao Zeng, Jing-Jing Wang, Zhen Liu, Guo-Qing Fan, Jin Li, Jian-Ping Cai

**Affiliations:** 1 The Key Laboratory of Geriatrics, Beijing Institute of Geriatrics, Institute of Geriatric Medicine, Chinese Academy of Medical Sciences, Beijing Hospital/National Center of Gerontology of National Health Commission, Beijing 100730, China; vita1023@163.com (Y.Z.); daisyqingyu@outlook.com (Q.-Y.W.); jane_computer@sina.com (Z.L.); missyueying@163.com (G.-Q.F.); niyani88@126.com (J.L.); 2 Graduate School of Peking Union Medical College, Beijing 100730, China; 3 Peking University Fifth School of Clinical Medicine, Beijing Hospital/National Center of Gerontology of National Health Commission, Beijing 100730, China; zengtiancai@sina.com; 4 Department of Clinical Laboratory, Henan Provincial People’s Hospital, People’s Hospital of Zhengzhou University, Zhengzhou 450066, China; 15133919987@163.com

**Keywords:** Western dietary pattern, metabolic disorders, pancreas, islets, single-cell RNA sequencing analysis

## Abstract

Long-term consumption of a Western diet is a major cause of type 2 diabetes mellitus (T2DM). However, the effects of diet on pancreatic structure and function remain unclear. Rats fed a high-fat, high-fructose (HFHF) diet were compared with rats fed a normal diet for 3 and 18 months. Plasma biochemical parameters and inflammatory factors were used to reflect metabolic profile and inflammatory status. The rats developed metabolic disorders, and the size of the islets in the pancreas increased after 3 months of HFHF treatment but decreased and became irregular after 18 months. Fasting insulin, C-peptide, proinsulin, and intact proinsulin levels were significantly higher in the HFHF group than those in the age-matched controls. Plasmatic oxidative parameters and nucleic acid oxidation markers (8-oxo-Gsn and 8-oxo-dGsn) became elevated before inflammatory factors, suggesting that the HFHF diet increased the degree of oxidative stress before affecting inflammation. Single-cell RNA sequencing also verified that the transcriptional level of oxidoreductase changed differently in islet subpopulations with aging and long-term HFHF diet. We demonstrated that long-term HFHF diet and aging-associated structural and transcriptomic changes that underlie pancreatic islet functional decay is a possible underlying mechanism of T2DM, and our study could provide new insights to prevent the development of diet-induced T2DM.

## 1. Introduction

Diabetes mellitus, also known as diabetes, is a serious metabolic disorder with multiple etiologies characterized by disturbances in carbohydrate, fat, and protein metabolism [1,2]. It is estimated that diabetes causes 11.3% of global deaths annually [3]. Overall, 4.2 million people in the 20–79-year age group died of diabetes in 2019 [3]. The increasing mortality associated with diabetes demonstrates the urgency to explore the pathogenesis of the disease.

The WHO proposed several dietary practices according to the confirmed correlation between obesity and type 2 diabetes mellitus (T2DM) risk [1,4]. The consumption of high amounts of fat and refined carbohydrates in the diet may be risk factors for dyslipidemia [5], obesity [6,7,8], insulin resistance [9], and heart disease [10,11]. In addition, many cross-sectional studies and meta-analyses have reported a positive correlation between strong adherence to a Western dietary pattern and an increased risk of T2DM [12,13,14,15,16,17,18,19,20,21,22,23]. However, the target organs and pathogenic mechanisms of diet in T2DM remain unclear.

In 2001, Brownlee first proposed that oxidative stress (OS) may play a major role in the pathophysiology of diabetes and its complications [24]. OS is caused by an imbalance between the production of reactive oxygen species (ROS) and the activity of the antioxidant defense system [25]. Epidemiological studies have also confirmed the elevation of OS biomarker levels in T2DM compared with those in healthy conditions, including antioxidant enzyme activity, lipid peroxidation, and nucleic acid oxidation markers. In a study that included 80 T2DM patients and 79 apparently healthy controls, the ferric reducing ability of plasma, γ-glutamyltransferase, and plasma glutathione reductase (GR) levels were significantly higher in patients with diabetes than those in nondiabetic people [26]. Bandeira et al. found that the total superoxide dismutase (SOD) activity and lipid peroxidation levels were higher in diabetes patients than those in non-diabetes patients through their case–control study (in the 40 to 86 year old group). In further stratified groups, the rate of lipid peroxidation was significantly higher in both groups of diabetes patients (hypertensive and normotensive) than in the prediabetic groups and controls, revealing that increased lipid peroxidation is closely related to T2DM [27]. Furthermore, the levels of the commonly used nucleic acid oxidation markers 8-dihydroguanosine (8-oxo-Gsn) and 8-hydroxy-2-deoxyguanosine (8-oxo-dGsn) were also higher in T2DM patients than those in healthy controls. Broedbaek et al. found that the level of the RNA oxidation marker 8-oxo-Gsn in urine independently predicted diabetes-related mortality [28,29]. Our previous study also verified that 8-oxo-Gsn level in urine was correlated with diabetic nephropathy, and that 8-oxo-Gsn might be a sensitive marker of diabetic nephropathy [30]. However, the mechanisms underlying OS targets in T2DM remain unclear.

Many T2DM animal models, including spontaneous and induced diabetes models, have been developed [31]. These models include characteristics such as insulin deficiency, insulin resistance, and/or beta cell failure [31]. Among induced rodent models, streptozotocin- or alloxan-induced diabetic models are the most common [31,32]. Both chemicals act as cytotoxic glucose analogs that accumulate in pancreatic beta cells through glucose transporter 2 (GLUT2), causing selective destruction of islet beta cells [31,32]. Although these models are helpful for studying the pathogenesis of diabetes, they are not in line with the internal environmental changes induced by long-term dietary treatments. Through the administration of a combination of fructose and fat, Lozano et al. first developed an ideal model for researching the effect of diet on the development of T2DM [33]. They noted marked hepatic vacuolar degeneration and increased ROS levels in the liver and vascular system of diabetic rats that had been fed a high-fat, high-fructose (HFHF) diet [33]. Dal et al. [34] and Werf et al. [35] found that diabetes symptoms and increased levels of ROS in the liver and vascular system induced by HFHF diet were partially corrected by supplementing the antioxidant-rich foods, such as cherries and red cabbages. Of note, existing studies have mainly focused on the HFHF diet-induced impairment of the liver, vascular system, cognition, and retina [36,37,38,39,40]. As an essential organ for sensing blood glucose concentrations and synthesizing and releasing glucose-regulating hormones, islets in the pancreas play a vital part in blood glucose homeostasis [41,42]. However, the effects of an HFHF diet on pancreatic structure and function, as well as their potential mechanisms, remain unclear.

Therefore, the present study explored the impact of a long-term HFHF diet on the rat pancreas. An 18 month HFHF diet was used to trigger the onset of T2DM in rats, which was confirmed by assessing metabolic, oxidative, and inflammatory parameters. After observing the changes in pancreatic structure and function, we used single-cell RNA-sequencing (scRNA-seq) to explore the distribution and transcriptomic patterns in pancreatic cells and islet cells. We decoded long-term HFHF diet and aging-associated structural and transcriptomic changes that underlie pancreatic islet functional decay at single-cell resolution and indicated that inflammation occurs after oxidative stress, suggesting a possible underlying mechanism of T2DM and providing new insights to prevent the development of diet-induced T2DM.

## 2. Materials and Methods

### 2.1. Ethics Statement

The animal use protocol was reviewed and approved by the Animal Ethics and Welfare Committee of Beijing Stomatological Hospital (Approval No. KQYY-202103-001). All efforts were made to minimize animal suffering and to reduce the number of animals used.

### 2.2. Animals and Induction of Diabetes

Forty male Wistar rats (male; 2 months old; 280.75 ± 8.19 g; SPF (Beijing Biotechnology Co., Ltd., Beijing, China), were fed in a temperature-controlled room with a 12 h light/dark cycle and ad libitum access to water and food. After 1 week, eight rats were randomly selected and sacrificed (baseline), and the other rats were then randomly divided into two groups of 16 rats each (control and HFHF groups). The control and HFHF groups were fed a normal diet and HFHF diet (SPF (Beijing) Biotechnology Co., Ltd.), respectively, as previously reported [33]. The primary ingredients of each diet are shown in Table S1.

**Figure 1:**
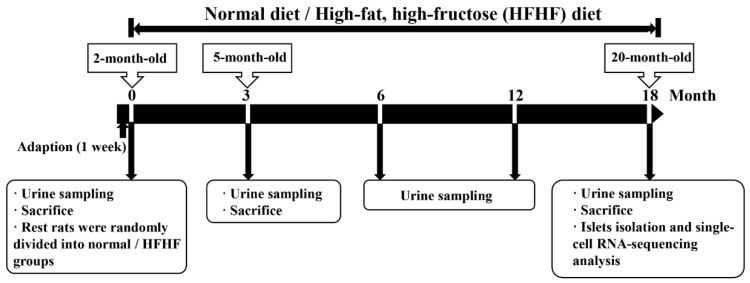
The study outline.

Eight rats per group were sacrificed 3 months after the start of the administration of each diet. The rats were intraperitoneally anesthetized using Zoletil^®^50 (Boehringer Ingelheim, Ingelheim, Rhein, Germany). Blood was collected from the heart, and plasma was stored at −80 °C for subsequent analysis. All tissues were rinsed, weighed, and embedded in Tissue-Tek^®^ Optimal Cutting Temperature compound (Sakura Finetek USA, Torrance, CA, USA) or frozen in liquid nitrogen. Spontaneous metabolism was assessed using standard rodent metabolic cages, and then 24 h urine was collected. After 18 months of feeding, six rats in each group were anesthetized and sacrificed according to the above procedures. The remaining two rats per group had their islets isolated for single-cell RNA sequencing analysis. The study outline is shown in Figure 1.

### 2.3. Metabolic, Inflammatory, and Oxidative Parameter Analyses

#### 2.3.1. Glucose Measurement

Blood glucose levels were evaluated on the basis of the fasting glucose value (OneTouch Ultra Easy^®^ glucometer; Johnson & Johnson, New Brunswick, NJ, USA) and intraperitoneal glucose tolerance test (IpGTT). A 20% glucose solution was injected intraperitoneally in rats at a 2 g/kg dose. A glucometer measured the caudal capillary glycemia of rats at 0, 15, 30, 60, and 120 min after injection.

#### 2.3.2. Plasmatic Metabolic and Oxidative Parameters Assessments

All procedures for determining glycated serum protein (GSP; A037-2-1; Nanjing Jiancheng Bioengineering Institute, Nanjing, China), pyruvic acid (A081), glucose (A154-1-1), total triglycerides (TG; A110-1-1), total cholesterol (T-CHO; A111-1-1), low-density lipoprotein cholesterol (LDL-C; A113-1-1), SOD (A001-3), xanthine oxidase (XOD; A002-1-1), total antioxidant capacity (T-AOC; A05-3-1), lactate dehydrogenase (LDH; A020-2), T-GSH (A061-1), GR (A062-1-1), hydrogen peroxide (H_2_O_2_; A064-1-1), and lipid hydroperoxide (LPO; A106-1) were performed according to the manufacturer’s instructions (Nanjing Jiancheng Bioengineering Institute, Nanjing, China).

#### 2.3.3. Detection of 8-oxo-Gsn and 8-oxo-dGsn in Urine

First, 100 μL of urine was mixed with 400 μL of 30% methanol (Sigma-Aldrich, St. Louis, MO, USA) solution to acquire a 1:5 diluted urine sample for oxidized guanine nucleoside analysis. Then, 10 μL of 80 pg/μL [_15_N^5^] 8-hydroxy-2-deoxyguanosine (8-oxo-dGsn; Cambridge Isotope Laboratories, Andover, MA, USA) and 10 μL of 80 pg/μL [_15_N^2^_13_C^1^] 8-dihydroguanosine (8-oxo-Gsn; Toronto Research Chemicals, Toronto, ON, Canada) were added as internal controls and centrifuged at 12,000× *g* for 15 min at 4 °C.

Next, 100 μL of supernatant was loaded in the liquid chromatograph with tandem mass spectrometry (LC–MS/MS) (Agilent Technologies, Santa Clara, CA, USA) for the analysis of 8-oxo-dGsn and 8-oxo-Gsn [30]. The mobile phase consisted of 20 mM ammonium acetate (Thermo Fisher Scientific, Waltham, MA, USA) (A) and 100% methanol (B), which was added in a Waters Sunfire C18 column (5 μm, 4.6 × 250 mm). Gradient elution was performed from 0 to 6.0 min with 8% B at 0.8 mL/min, from 6.0 to 6.01 min changing from 8% B to 50% B at 1 mL/min (sixth curve), from 6.01 to 8 min in 50% B at 1 mL/min, from 8.0 to 8.01 min changing from 50% B to 8% B (sixth curve), and then from 8.01 to 10.0 min in 8% B at 0.8 mL/min (sixth curve). The injection volume was 20 μL, and the UV detection wavelength was 233 nm. The final concentrations of 8-oxo-dGsn and 8-oxo-Gsn in urine were calculated on the standard curve.

#### 2.3.4. Plasmatic Inflammatory Parameters

Interleukin (IL)-1β, IL-6, tumor necrosis factor (TNF)-α, vascular endothelial growth factor (VEGF), intercellular cell adhesion molecule (ICAM)-1, and monocyte chemotactic protein (MCP)-1 were detected using enzyme-linked immunosorbent assay (ELISA) at the Nanjing Jiancheng Bioengineering Institute.

### 2.4. Histological Studies

To estimate histological changes, embedded pancreatic tissues were sectioned to a thickness of 8 μm for hematoxylin/eosin (HE) staining (G1121; Beijing Solarbio Science & Technology Co., Ltd., Beijing, China), Masson’s trichrome staining (G1346; Beijing Solarbio Science s& Technology Co., Ltd., Beijing, China), and immunofluorescent staining. Mouse anti-insulin (rat, 1:500, ab181547; Abcam, Waltham, MA, USA), rabbit anti-glucagon (rabbit, 1:150, 15954-1-AP; Proteintech, Rosemont, IL, USA), and the corresponding secondary antibodies (donkey anti-rabbit IgG (H + L) Highly Cross-Adsorbed Secondary Antibody, Alexa Fluor 555; donkey anti-mouse IgG (H + L) Highly Cross-Adsorbed Secondary Antibody, Alexa Fluor 488(REF.A21202); Life Technologies, Carlsbad, CA, USA) were used to detect alpha and beta islet cells. As described in Chansela et al.’s study, the islets could be categorized as small (<10,000 μm^2^), medium (10,000–50,000 μm^2^), large (50,000–100,000 μm^2^), and extra large (>100,000 μm^2^) on the basis of the total area of islets [43]. The islet density is calculated as follows: the number of each islet size/total number of islets ×100 [43].

### 2.5. Pancreas-Specific Endocrine Protein Expression in Plasma

To evaluate the function of the pancreatic islets, fasting plasma insulin (Mercodia High Range Rat Insulin ELISA, Lot10-1145-01; Mercodia, Uppsala, Sweden) and C-peptide levels (Mercodia Rat C-peptide ELISA, Lot10-1172-01; Mercodia) were tested using a double-antibody sandwich enzyme-linked immunoassay. Proinsulin (Mercodia High Range Rat Proinsulin ELISA, Lot 10-1232-01; Mercodia) and intact proinsulin (Mouse/Rat Intact Proinsulin Assay Kit, No. 27706; Immuno-Biological Laboratories Co., Ltd., Naka, Fujioka-Shi, Japan) were examined according to the manufacturer’s instructions.

### 2.6. 10X Genomics Single-Cell RNA Sequencing Analysis of Pancreas in 18 Month HFHF Rats

#### 2.6.1. Preparation of Single-Cell Suspension

Pancreases of two rats per group (baseline, HFHF, and control groups after 18 months of feeding) were subjected to scRNA-seq. The islets were isolated as previously reported [44,45]. Briefly, precooled 0.5 mg/mL collagenase P solution was injected into the common bile duct to expand the pancreas, and then the pancreas was quickly extracted and transferred into a digestion bottle. After incubation at 38 °C for 10 min, the suspension was centrifuged at 1200× *g* for 2–3 min at 4 °C. The precipitate was washed twice with Hanks’ solution and resuspended in Histopaque-1077. After gently adding RPMI 1640 (containing 10% fetal bovine serum) solution to the liquid surface, the tube was centrifuged at 3000× *g* for 20 min at 4 °C. Granules scattered between the interfaces were collected and resuspended in 1 mL TrypLE (12604013; Thermo Fisher Scientific) for further digestion. DAPI staining was used to evaluate cell viability (Count Star, TANON SCIENCE & TECHONLOGY CO, Shanghai, China). When cell viability reached 80% and the proportion of doublets was less than 20%, an estimated 10,000 single cells per sample were suspended in phosphate-buffered saline solution (containing 0.04% bovine serum albumin).

#### 2.6.2. Cell Capture and cDNA Synthesis

Cell suspensions were loaded on a chromium single-cell controller (10× Genomics, San Francisco, CA, USA) to generate single-cell gel beads in emulsion (GEMs). A Chromium Next GEM Single Cell 3ʹ GEM, Library & Gel Bead Kit v3.1 (1000121; 10× Genomics, Pleasanton, CA, USA) and Single-Cell B Chip Kit (1000127; 10× Genomics) were used according to the manufacturer’s protocol. As previously described, single-cell droplet collection, cDNA amplification, and sequencing library preparation were performed [46,47]. The cDNA libraries were sequenced on an Illumina Novaseq6000 sequencer with a sequencing depth of at least 30,000 reads per cell and a pair end of 150 bp (PE150; Capitalbio Technology Corporation, Beijing, China).

#### 2.6.3. scRNA-Seq Data Preprocessing

Data were analyzed by Capitalbio Technology Corporation (Beijing, China). Cell Ranger software (ver 6.1.2 accessed on 25 October 2021; https://support.10xgenomics.com/single-cell-gene-expression/software/downloads/latest) was used to perform alignment, filtering, barcode counting, and unique molecular identifier (UMI) counting and then to generate a feature barcode matrix and determine clusters. Cells whose gene number was <200, whose gene number was ranked in the top 1%, or whose mitochondrial gene ratio was >25% were regarded as abnormal and filtered out. Data were then loaded into Seurat 3.0 (accessed on 6 June 2019; http://satijalab.org/seurat) without normalization, scaling, or centering. In addition to the expression data, metadata for each cell were collected, including clone identity, cell-cycle phase, and sample source. Highly variable genes were identified and used as inputs for dimensionality reduction using principal component analysis. The resulting principal components (PCs) and correlated genes were examined to determine the number of components included in the downstream analysis. T-distributed stochastic neighbor embedding and uniform manifold approximation and projection (UMAP) plots (based on the first 10 PCs) were used to visualize the cells in a two-dimensional space. Gene Ontology (GO) enrichment analysis was performed using the Rstudio (Version 4.1.2) software program with Benjamini–Hochberg multiple testing adjustment, which was based on all differentially expressed genes (*p*-adj < 0.05, avg_log_2_FC > 0.585).

### 2.7. Statistical Analyses

Results are expressed as the mean ± standard deviation (SD) or median (interquartile range; IQR). Statistical analyses were performed using Student’s *t*-test, Mann–Whitney U test for unpaired data (SPSS^®^, 22.0, Chicago, IL, USA), or two-way analysis of variance and Tukey’s post hoc test (GraphPad Prism^®^, 8.3.1, San Diego, CA, USA). Statistical significance was set at *p* < 0.05.

## 3. Results

### 3.1. Long-Term HFHF Diet Induced Diabetes

**Figure 2:**
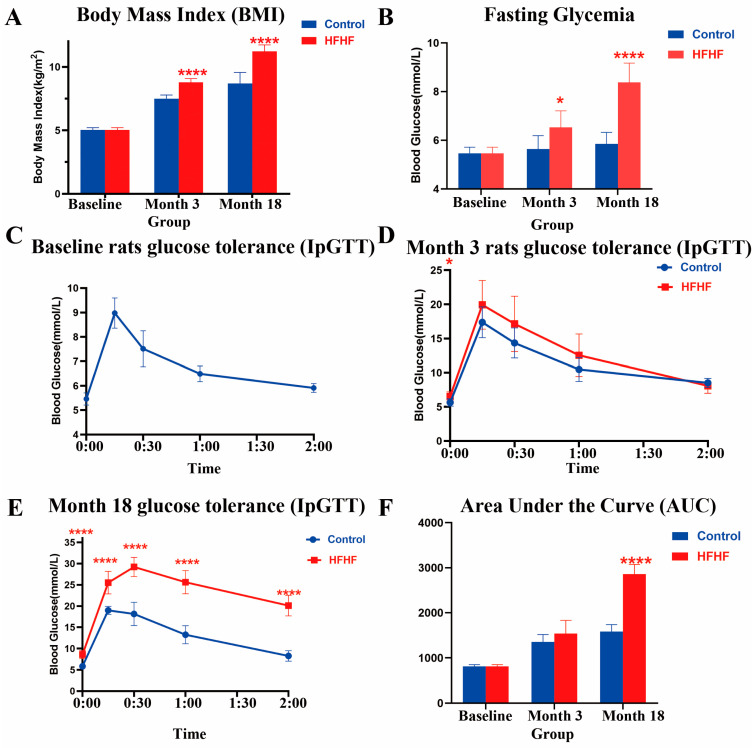
BMI, fasting glucose, and glucose tolerance measured in this study. (**A**) The body mass index (BMI) in this study for the normal diet (control, blue) and high-fat, high-fructose diet (HFHF, red) groups. (**B**) Evolution of fasting glycemia. (**C**–**E**) Evolution of glucose tolerance measured via the intraperitoneal glucose tolerance test (IpGTT) in all groups at the beginning (baseline) and after 3 (month 3) and 18 (month 18) months of feeding. (**F**) The area under the curve (AUC) during the IpGTT. Results are shown as the mean ± SD. * Significant results versus the age-matched control group: * *p* < 0.05, **** *p* < 0.0001.

Compared to those of the control group, after 3 months of HFHF treatment, the HFHF diet induced a sharp increase in body weight (*p* < 0.0001) and body mass index (BMI; *p* < 0.0001) in the rats of the HFHF group (Table S2, Figure 2A and Figure S1). Fasting glycemia increased significantly between groups (*p* < 0.05) (Figure 2B). Lipid-related biochemical parameters increased in the HFHF group (Table S2). Obvious vacuolar degeneration in the livers of HFHF diet-fed rats was also observed (Figure S2).

After 18 months of treatment, the HFHF group exhibited more severe metabolic disorders, particularly dyslipidemia and hyperglycemia. Compared to the age-matched control group, the fasting glycemia level (*p* < 0.0001) and AUC of the IpGTT test (*p* < 0.0001) were significantly higher in the HFHF group (Figure 2). Lipid droplets showed further accumulation, and hepatic fibrosis was observed in the liver of HFHF rats (Figure S2). These findings suggest that long-term HFHF induces T2DM.

### 3.2. HFHF Diet Influenced Islet Cell Function and Structure

Insulin, secreted by islet cells, is the essential hormone responsible for maintaining glucose homeostasis [48]. Proinsulin, a precursor molecule of insulin, is intracellularly cleaved by prohormone convertases, which hydrolyze positions 32–33 and 65–66 in proinsulin, and the carboxypeptidase E, which removes the basic residues 64–65 and 31–32 to form insulin and C-peptide [41,49,50,51,52]. Therefore, the total proinsulin consists of several partially hydrolyzed forms of proinsulin intermediate (32–33 split proinsulin, 65–66 split proinsulin, des 64–65 proinsulin, and des 31–32 proinsulin) and intact proinsulin, which is the unhydrolyzed form of proinsulin [51,52,53,54]. Hence, the detection of total proinsulin and intact proinsulin could reflect the hormone-processing ability of islet cells.

**Figure 3:**
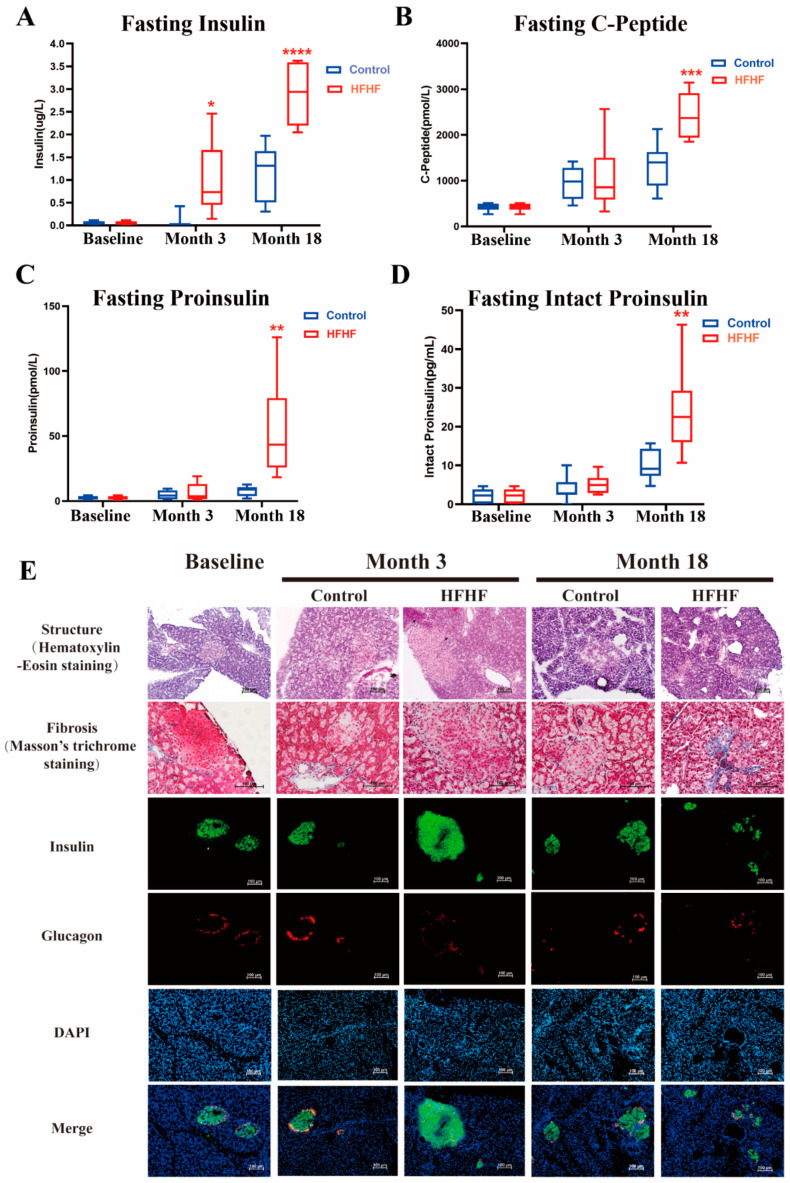
Structural and function changes of the pancreas in this study. (**A**–**D**) Fasting plasmatic insulin, C-peptide, proinsulin, and intact proinsulin levels during the study for the normal diet (control, blue) and high-fat, high-fructose diet (HFHF, red) groups at the beginning (baseline) and after 3 (month 3) and 18 (month 3) months of feeding. Results are shown as the mean ± SD. * Significant results versus the age-matched control group: * *p* < 0.05, ** *p* < 0.01, *** *p* < 0.001, **** *p* < 0.0001. (**E**) Changes in islets were visualized using hematoxylin/eosin staining, Masson’s Trichrome staining, and insulin and glucagon immunofluorescence staining at the beginning (baseline) and after 3 (month 3) and 18 (month 18) months of normal (control) and high-fat, high-fructose (HFHF) diet feeding. Scale bar = 100 μm.

To assess the functional consequences of an HFHF diet on the pancreas, levels of the above hormones were measured using ELISA (Figure 3A–D). After 3 months of receiving the appropriate diet, the fasting insulin level of HFHF rats was higher than that of the age-matched control group, whereas there were no differences in C-peptide, proinsulin, and intact proinsulin. After 18 months, the levels of these four hormones were significantly higher in the HFHF group than in the control group. Furthermore, during the IpGTT, the insulin, C-peptide, and proinsulin levels were also higher in the HFHF group than in the control group at each timepoint (Figure S3).

The islet structure was examined using HE staining. As shown in Figure 3E and Figure S4, the size of islets in the pancreas of the HFHF group rats increased after 3 months. However, after 18 months of feeding, islet size decreased, and the morphology became irregular. This phenomenon was confirmed by immunofluorescence staining (Figure S4). At the same time, the pancreatic tissue of rats in this group was found to have been infiltrated by a large number of adipocytes (Figure S5).

### 3.3. HFHF Diet Increased the Plasmatic Degree of Oxidation

**Figure 4:**
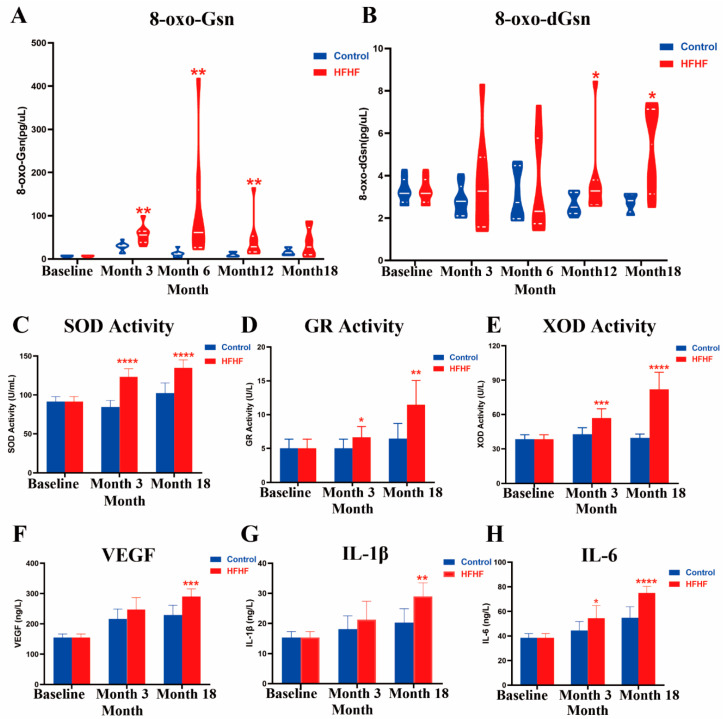
Changes in oxidation and inflammation levels during this study. Evolution of 8-oxo-Gsn (**A**) and 8-oxo-dGsn (**B**) during the study for the normal diet (control, blue) and high-fat, high-fructose diet (HFHF, red) groups. Results are shown as the median (interquartile range). (**C**,**D**) Effects of diet on SOD (**C**), GR (**D**), and XOD (**E**) activity at the beginning (baseline) and after 3 (month 3) and 18 (month 18) months of control and HFHF diet. (**F**–**H**). Effects of diet on VEGF (**F**), IL-1β (**G**), and IL-6 (**H**) during the study at the beginning (baseline) and after 3 (month 3) and 18 (month 18) months of treatments. Results are shown as the mean ± SD. * Significant results versus the age-matched control group: * *p* < 0.05, ** *p* < 0.01, *** *p* < 0.001, **** *p* < 0.0001.

As shown in Figure 4A, 8-oxo-Gsn levels were significantly higher in the urine of the HFHF group rats than that of the age-matched control group (3 months, *p* < 0.01; 6 months, *p* < 0.01; 12 months, *p* < 0.01; 18 months, *p* = 0.4). A similar trend was observed in urinal 8-oxo-dGsn detection. After 12 months of HFHF treatment, the level of 8-oxo-dGsn in the HFHF group increased significantly compared to the age-matched control group (12 months, *p* < 0.05; 18 months, *p* < 0.05) (Figure 4B), while the amount of 8-oxo-dGsn in the normal diet group remained unchanged during the study. Thus, long-term consumption of an HFHF diet induces RNA and DNA oxidation, suggesting the presence of an over-oxidative environment.

After administering the HFHF diet for 3 months, the levels of plasma oxidative parameters were significantly higher than those in the age-matched control group (Figure 4C–E, Table S2). After another 15 months of the HFHF diet, the levels of antioxidant enzyme activity (GR and XOD) and T-AOC in the HFHF group were twice those of the control group, suggesting that plasma oxidation was further intensified (Figure 4D,E, Table S2). Moreover, the expression of GSH, which acts as a ROS scavenger, decreased gradually (Table S2). These results suggest that the degree of OS in rats increased with consumption of the HFHF diet.

### 3.4. HFHF Diet Caused Inflammation

After feeding an HFHF diet for 3 months, compared with the age-matched group, there was no significant change in the plasma inflammation indices, except for IL-6 (*p* < 0.05) (Figure 4F–H, Table S2). Importantly, after 18 months, all inflammatory factors, including IL-1β (*p* < 0.01), IL-6 (*p* < 0.0001), MCP-1 (*p* < 0.0001), TNF-α (*p* < 0.05), VEGF (*p* < 0.001), and ICAM1 (*p* < 0.05) showed significant differences, indicating that the long-term HFHF diet induced an elevation in inflammation.

### 3.5. Identification and Transcriptomic Pattern of Pancreatic Cells from Single-Cell Levels in HFHF Rats after 18 Months

Six rats divided into baseline (young), HFHF (HFHF-old), and age-matched control (old) groups after 18 months of treatment were sacrificed, and their pancreases were isolated for scRNA-seq. A total of 78,024 cells were processed and qualified. After filtering the abnormal cells, data from 76,383 cells were loaded into Seurat for subsequent analysis (Figure S6F).

**Figure 5:**
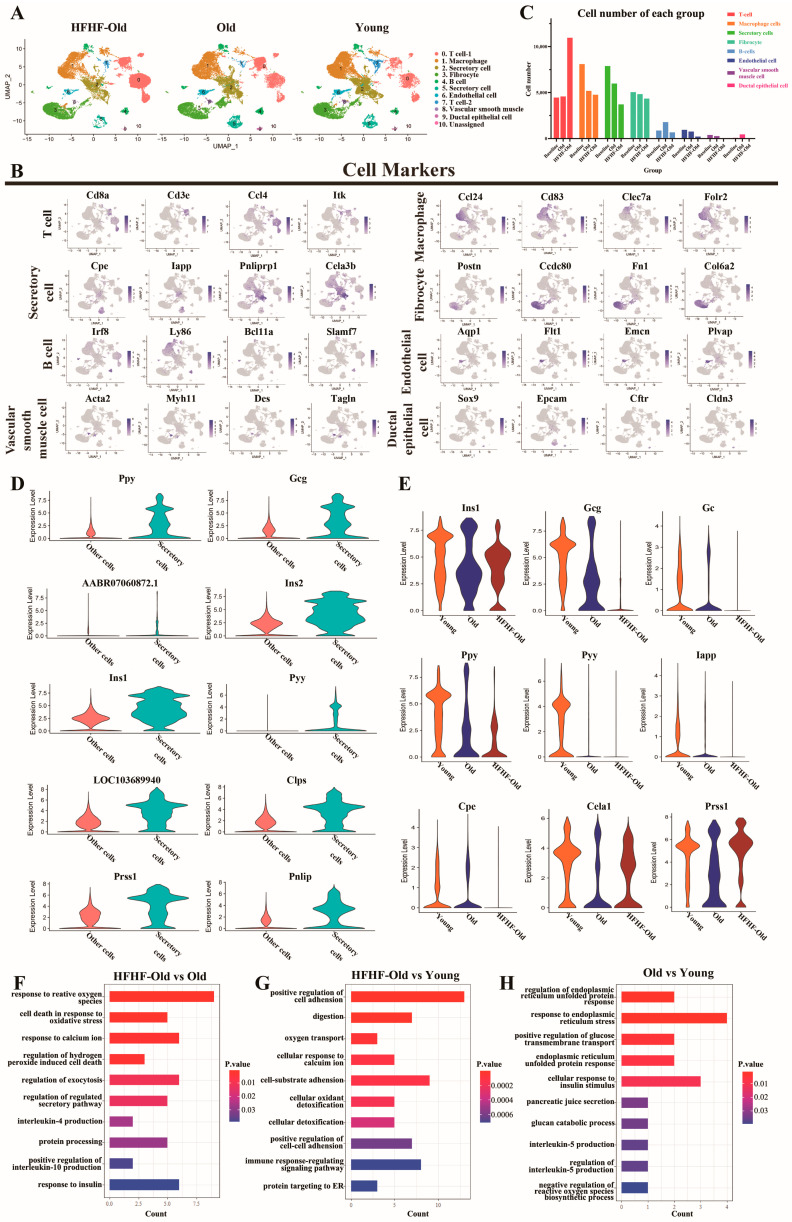
Distinct cell populations with transcriptional signatures in pancreatic secretory cells determined by a scRNA-Seq analysis of diabetic rats. (**A**) UMAP plot characterizing the differences in pancreatic cell distribution at the beginning (young) and after 18 months (HFHF-old, old) of diet feeding. Each point represents one cell. The distance between the two points is determined by the Pearson correlation of the 500 most highly expressed genes in each cell. Cell identity is recognized by the expression of marker genes. (**B**) UMAP plot of the marker gene expression in different cell types. (**C**) The number of different pancreatic cells at the beginning (young) and after 18 months (HFHF-old, old) of diet feeding. (**D**) Violin diagram of top 10 genes highly expressed in pancreatic secretory cells. (**E**) Violin diagram of secretory function-related gene expression in secretory cells among HFHF-old (HFHF, 18 months), old (control, 18 months), and young (baseline) groups. (**F**–**H**) Gene Ontology terms of upregulated enrichment obtained in secretory cells after a pairwise comparison of three groups (*p* < 0.05).

Unsupervised clustering analysis separated the cells into 10 clusters (Figure 5A and Figure S7A). Specific cell markers were used to recognize identities of cell clusters, including *Cd3e* and *Cd8a* (T cells), *Ccl24* and *Cd83* (macrophage cells), *Cpe* and *Pnliprp1* (secretory cells), *Postn* and *Ccdc80* (fibroblast cells), *Irf8* and *Ly86* (B cells), *Aqp1* and *Flt1* (endothelial cells), *Acta2* and *Myh11* (vascular smooth muscle cells), and *Sox9* and *EpCAM* (ductal epithelial cells) (Figure 5B). The number of cell types in the pancreas differed among the three groups (Figure 5A,C). GO analysis indicated that inflammation and immune-related pathways were significantly enriched in the HFHF-old group compared to the old group (Figure S7C–E), which was consistent with a significant increase in the T-cell count (Figure 5C). These observations indicate that long-term HFHF diet altered the pancreatic cellular composition.

### 3.6. Long-Term HFHF Diet Affected the Expression of Characteristic Genes and Enriched Oxidative and Inflammatory Pathways in Secretory Cells

We then explored the changes in the expression of characteristic genes in secretory cells caused by the long-term HFHF diet and aging. As the cells in clusters 2 and 5 were classified as secretory cells (Figure 5A,B), we combined the two clusters and compared them with other types of cells. The top 10 differentially expressed genes in secretory cells are shown in Figure 5D, most of which were related to the synthesis of glucose-regulating hormones and pancreatic proteases. GO analysis indicated that peptide hormone secretion- and digestion-related pathways were significantly enriched in the secretory cells (Figure S8). We found different expression patterns among the genes related to hormone synthesis in the three experimental groups. As shown in Figure 5E, the expression of genes related to glucose-regulating hormone synthesis decreased in the HFHF-old group, including *Ins1*, *Gcg*, *Ppy*, and *Pyy*. The total number of secretory cells also confirmed this among the groups, which showed that secretory cells number decreased in the old group, and the long-term HFHF diet exacerbated this decline (Figure 5C). In addition, GO analysis indicated that OS-related pathways were enriched in the secretory cell clusters of the HFHF-old group, while endoplasmic reticulum unfolded protein response-related pathways were mainly enriched in the old group (Figure 5F–H). Furthermore, inflammatory pathways were detected in the HFHF-old and old groups (Figure 5F–H). All of the above results verified that endoplasmic reticulum stress induced by unfolded proteins increases with age. A long-term HFHF diet downregulated the expression of glucose-regulating hormones and secretory cell numbers, while it potentially upregulated oxidative and inflammation-related pathways in pancreatic secretory cells.

### 3.7. The Long-Term HFHF Diet Affected the Number of Islet Cells and the Expression of Characteristic Genes

**Figure 6:**
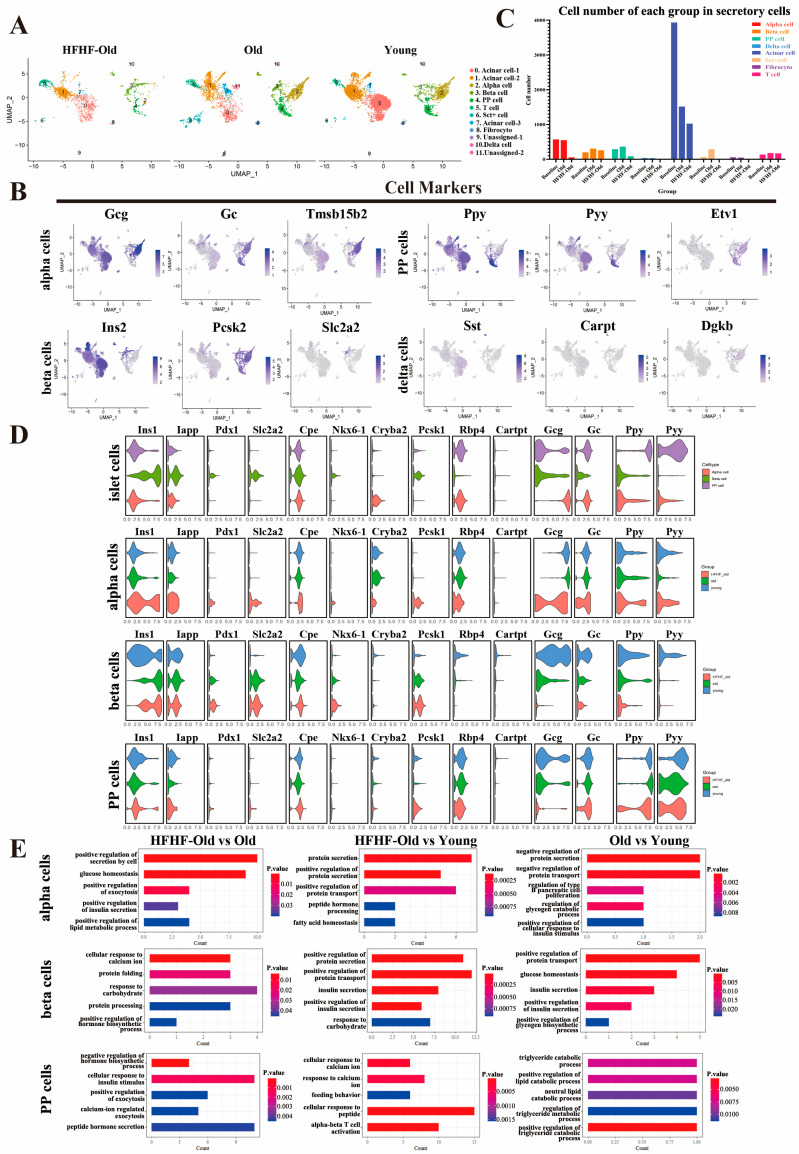
Distinct cell populations with transcriptional signatures in secreting islet cells. (**A**) UMAP plot characterizing the differences in secretory cell subpopulation distribution at the beginning (young) and after 18 months (HFHF-old, old) of diet feeding. Each point represents one cell. The distance between the two points is determined by the Pearson correlation of the 500 most highly expressed genes in each cell. Cell identity is recognized by the expression of marker genes. (**B**) UMAP plot of the marker gene expression in four types of islet cells. (**C**) The number of different pancreatic secretory cells at the beginning (young) and after 18 months (HFHF-old, old) of diet feeding. (**D**) Violin diagram characterizing the marker genes expression level in islet alpha, beta, and PP cells at the beginning (young) and after 18 months (HFHF-old, old) of diet feeding. (**E**) GO terms of upregulated secretory function-related enrichment obtained in islet alpha, beta, and PP cells after pairwise comparison of three groups (*p* < 0.05).

Four major endocrine cell types in islets and acinar cells were identified using specific cell markers: *Gcg* and *Gc* (alpha cells), *Ins1* and *Pcsk2* (beta cells), *Ppy* and *Pyy* (polypeptide (PP) cells), *Sst* (delta cell), and *Prss2* and *Cela1* (acinar cells) (Figure 6A,B and Figure S9A,B). The number of islet cells, especially alpha, PP, and delta cells, was significantly decreased in the HFHF-old group, whereas that in the old group barely changed (Figure 6C). The number of beta cells decreased slightly in the long-term HFHF group compared to that in the old group. Since only eight delta cells were obtained in the long-term HFHF group, which was too few to analyze the differential expressed genes between groups, these islet cells were not included in subsequent transcriptional and functional studies.

To further analyze the effects of long-term HFHF and the unavoidable aging on functional genes in islets, we compared the expression of several of the most common marker genes and transcriptional regulators of these three cells among the three experimental groups. Several known beta cell markers were also identified, including *Iapp* encoding islet amyloid polypeptide and *Slc2a2* (also known as *Glut2*) which is responsible for glucose uptake [55] (Figure 6D). During the aging process, the expression of major hormone-biosynthetic genes in islet cells, such as *Gcg* in alpha cells, *Ins1* in beta cells, and *Ppy* and *Pyy* in PP cells, was upregulated, but the long-term HFHF diet caused downregulation of these genes (Figure 6D). *Pdx1* (encoding pancreas/duodenum homeobox protein 1 and binding to the regulatory elements to upregulate insulin gene transcription [56]) and *Slc2a2* expression in beta cells was upregulated with aging, and the long-term HFHF diet further upregulated these genes. The expression of *Pdx1* and *Slc2a2* in alpha cells was also upregulated in the HFHF group. The long-term HFHF diet further exacerbated the downregulation of *Rbp4* (encoding retinol-binding protein 4, which is an adipokine involved in the development of obesity and insulin resistance [57]) expression in beta cells compared to the age-matched group. The expression of *Pcsk1*(encoding proprotein convertase subtilisin/kexin type 1, which has been identified in patients with early-onset obesity [58,59]) increased in alpha cells with a long-term HFHF diet. In addition, the expression of *Ins1* in alpha cells was increased in the HFHF group (Figure 6D). We further performed GO analysis to confirm functional changes in different islet cells, and the results showed that the long-term HFHF diet altered hormone secretion function as a positive regulation of protein secretion, while cellular response to calcium ion and protein processing pathways were significantly enriched (Figure 6E). In alpha cells, positive regulation of secretion, exocytosis, and peptide hormone processing were enriched in the HFHF-old group, suggesting that a long-term HFHF diet might increase function-related hormone processing and secretion in alpha cells. In beta cells, the positive regulation of protein secretion, insulin secretion, and positive regulation biosynthetic processes were also enriched in the HFHF-old and age-matched old groups. In PP cells, the protein hormone secretion and exocytosis pathways were enriched only in the HFHF-old group. Long-term HFHF treatment might downregulate the synthesis of glucose-regulating hormones but might increase hormone secretion in subpopulations of islets.

### 3.8. Long-Term HFHF Diet Increased the Oxidative Stress Level in Islet Cells

**Figure 7:**
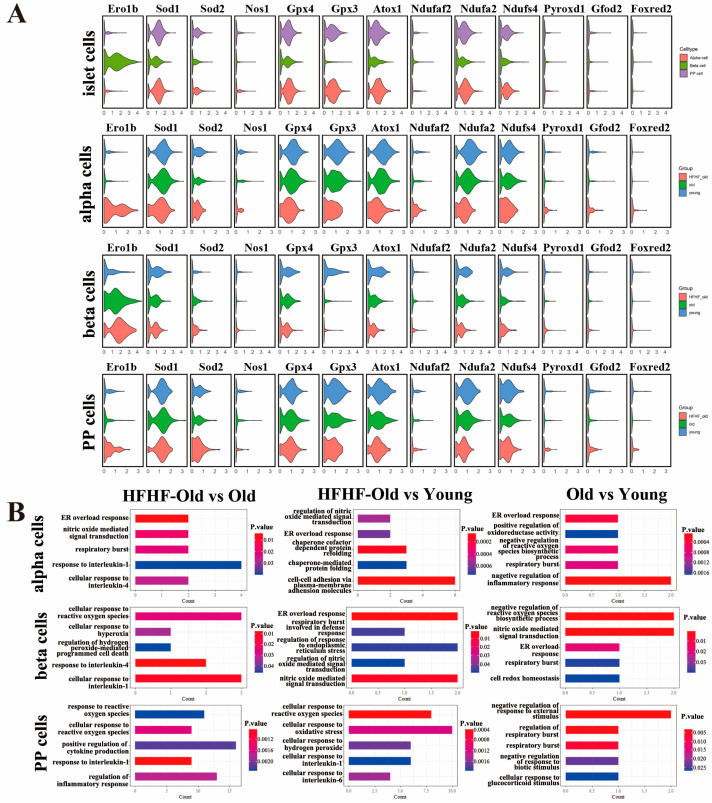
Distinct expression of oxidative-related oxidoreductase genes and Gene Ontology (GO) analysis findings among three groups. (**A**) Violin diagram characterizing the expression of oxidoreductase genes in islet alpha, beta, and PP cells at the beginning (young) and after 18 months (HFHF-old, old) of diet feeding. (**B**) GO terms of upregulated OS and inflammation-related enrichment obtained in islet alpha, beta, and PP cells after a pairwise comparison of three groups (*p* < 0.05).

As the blood oxidative parameters were significantly increased in the HFHF group, we further investigated the oxidation-related genes and pathways that changed within islet subpopulations. The oxidoreductase gene expression differed among islet cell types (Figure 7A). The expression of *Atox1* (antioxidant 1 copper chaperone) and *Ndufs4* (ubiquinone oxidoreductase subunit S4) in alpha, beta, and PP cells gradually decreased with age, and the long-term HFHF diet further exacerbated the downregulation of these genes. The expression level of *Pyroxd1* (pyridine nucleotide-disulfide oxidoreductase domain 1) decreased in the old group, while it recovered in the HFHF-old group. Among alpha cells, *Ero1b* (endoplasmic reticulum oxidoreductase 1 beta) expression also decreased with age but was upregulated by the long-term HFHF diet. In contrast, in beta and PP cells, the expression of *Ero1b* was increased in the old group and further upregulated in the HFHF-old group. In alpha cells, the *Sod1* (superoxide dismutase 1) expression decreased with age, and long-term administration of an HFHF diet further exacerbated this decline. In beta cells, the expression of *Nos1* (nitric oxide synthase 1) was decreased in the old group, but the long-term HFHF diet induced partial recovery. In PP cells, the expression of *Gfod2* (glucose-fructose oxidoreductase domain containing 2) and *Foxred2* (FAD-dependent oxidoreductase domain containing 2) was higher in the HFHF-old group than in the old group. As the violin plots showed that a long-term HFHF diet increased the expression of various oxidoreductases in different subpopulations of islets, GO analyses were also proposed to determine changes in oxidative- and inflammation-related pathways in different islet cells. Oxidative-related biological processes were enriched in islet cells including beta and PP cells of HFHF-old group, such as response to reactive oxygen species biological processes and cellular response to oxidative stress compared with the old group (Figure 7B). However, negative regulation of reactive oxygen species was enriched in alpha and beta cells from the old group, suggesting that ROS could also be produced with normal aging. Combined with the slow change in plasma antioxidant parameters in the old group, this suggested that the level of ROS induced by aging was still within the normal range and did not cause OS. In addition, inflammation-related biological processes, including the response to IL-4 and IL-1, were only enriched in the HFHF-old group, suggesting that long-term HFHF diet caused inflammation in the alpha, beta, and PP cells of islets. On the basis of the above, a long-term HFHF diet changed the expression pattern of oxidoreductase genes and increased oxidation and inflammation in islet subpopulations.

## 4. Discussion

Various HFHF dietary patterns, which can induce diabetes in rats, have been reported [33,34,35,36,37,38,39,40,60]. However, the dose of fructose and fat administered to rodents in many studies was higher (50–60% of the diet) than that consumed by humans (10–15%) [61]. Therefore, we chose a model that more closely resembles the human diet (21.44% fat, 17.45% protein, 50.26% carbohydrate, and 20% fructose in water) [33]. Previous animal studies have shown that an HFHF diet induces persistent metabolic disorders and OS in pathogenesis, as well as hepatic and vascular complications [33,34,35]. We also found abnormal glucose and lipid metabolism in rats after 3 months of the HFHF diet, with significant abnormalities in glucose tolerance noted after 18 months. In our study, obvious vacuolar degeneration in the liver and an increase in oxidative parameters (such as GR, SOD, and 8-oxo-Gsn in urine) were also observed, consistent with previous human epidemiology [26,27,28,29] and animal [30,33,34,35] studies.

The function of islets is to secrete glucose-regulating hormones to maintain glucose homeostasis; however, this function is impaired in T2DM patients [48]. Therefore, we further explored changes in the structure and function of islets in a T2DM rat model induced by a long-term HFHF diet. After 3 months of an HFHF diet, the size of the islets increased. This morphological change was also verified in ovariectomized rats with an HFHF diet [43]. As only the level of insulin increased significantly and diabetes did not develop at this time, the change in islets might suggest that compensatory hyperplasia occurred. This result is consistent with previous research showing that beta-cell mass expansion is modulated indirectly by insulin via the nuclear hormone receptor peroxisome proliferator-activated receptor γ in response to obesity [62,63,64,65,66]. Notably, after 18 months of feeding, the islet volume decreased unexpectedly, and the morphology became irregular, which was consistent with the significant increases in fasting glucose levels, glucose tolerance, and levels of immature precursors of insulin. These results showed that an increase in islet mass occurred in the early compensatory phase in T2DM, while islet mass decreased in the late phase.

As the pancreatic structure and its secretory function were remarkably changed, scRNA-seq analysis was conducted to further explore changes in the cell composition and transcriptome patterns of pancreatic cells, secretory cells, and subpopulations of islet cells. A scRNA-seq analysis could help researchers to explore cell heterogeneity, identify cell states, and show changes in cell-type-specific genes during aging or disease development [67]. Previous studies have analyzed human islets via scRNA-seq, enabling a better understanding of islet pathology in aging and T2DM. Wang et al. found that alpha and beta cells from donors with T2DM had expression profiles with features observed in children, which indicated a partial dedifferentiation process [68]. Enge et al. found that an elevation of transcriptional noise and potential fate drift occurred in islet cells from elders [69]. Xin et al. identified 245 genes with disturbed expression in type 2 diabetes, 92% of which were not involved in islet cell function or growth in the previous study [70]. Rat, a popular animal model for studying T2DM, has a loosely dispersed mesenteric pancreas, which makes it difficult for researchers to collect and prepare single-cell suspensions for scRNA-seq studies. Thus, it would be worthwhile to explore methods and identify the characteristics and transcriptome patterns of rat islets and their subpopulations. In this study, cell-specific markers were used to identify secretory, immune, fibrocyte, endothelial, vascular smooth muscle, and ductal epithelial cells in the pancreas. We found that the secretory population decreased with age, and the long-term HFHF diet exacerbated this decline. Examination of the secretory cell subtypes showed that the number of islet cells, especially alpha, delta and PP cells, was significantly decreased in the HFHF-old group compared to the young group, while that of the old group was nearly unchanged. Among the three experimental groups, the expression of genes related to glucose-regulating hormone synthesis was upregulated during the aging process; however, the long-term HFHF diet caused downregulation. Nevertheless, GO analysis showed a positive regulation of hormone secretion induced by a long-term HFHF diet, while the positive regulation of protein secretion, exocytosis, peptide hormone processing, and positive regulation biosynthetic processes were enriched in islet cells of the HFHF group. These results suggest that a long-term HFHF diet may reduce the synthesis of glucose-regulating hormones while promoting the release of these hormones. Furthermore, the expression of *Ins1* in alpha cells increased with age and was further exacerbated by a long-term HFHF diet. This was confirmed by the increase in plasma insulin, proinsulin, and intact proinsulin levels, suggesting islet dysfunction.

Several studies have shown that the degree of oxidation increases in diabetic patients [24,26,27]. We also found elevated levels of plasma GR, SOD, XOD, GR, T-AOC, urine 8-oxo-Gsn, and 8-oxo-dGsn in rats fed an HFHF diet; however, the changes in the pancreas or islets were still not clear. Therefore, we used scRNA-seq to explore the transcriptome patterns of islet cells and their subgroups of oxidation-related genes and biological processes. In the present study, scRNA-seq showed that the long-term HFHF diet increased the degree of oxidation of secretory cells. GO analysis indicated that OS-related pathways were enriched in secretory cell clusters with the long-term HFHF diet, whereas the endoplasmic reticulum stress response induced by unfolded proteins was more significant during aging. In islet cells, different oxidoreductase gene expression in the HFHF-old group was upregulated in three subtypes of islet cells (alpha, beta, and PP cells), including *Pyroxd1*, *Ero1b*, *Nos1*, *Gfod2*, and *Foxred2*. Furthermore, compared with those of the old group, oxidative-related biological processes, such as the response to reactive oxygen species, were also enriched in the islet cells of the HFHF group. Moreover, the transcriptional level of oxidoreductase also changed in islet cells with aging; for example, *Sod1* decreased with age in alpha and beta cells, and a long-term HFHF diet further exacerbated this decline. All the above results verified that the long-term HFHF diet accelerated OS in the pancreas and islet subpopulations.

In addition, inflammatory pathways have also been regarded as the potential pathogenic mediators of overweight and DM [71,72]. Several reports support the notion that islet inflammatory processes are involved in the pathophysiological changes in T2DM [73]. We also found that inflammation levels were significantly increased in the long-term HFHF-treated rats. However, the cause of islet inflammation remains unclear [72]. We found that inflammation-related items, including the response to IL-4 and IL-1, were significantly enriched in alpha, beta, and PP cells of islets in the HFHF-old group according to GO analysis from the ScRNA-seq data. In addition, our plasma findings also confirmed that a long-term HFHF diet induced inflammation. After feeding the HFHF diet for 3 months, the levels of plasma oxidative parameters were significantly increased, while those of plasma inflammatory factors, including IL-1β, IL-6, MCP-1, VEGF, and ICAM1, were only changed after 18 months of feeding. The results showed that inflammation occurred after oxidative stress, suggesting that oxidative stress might be the cause of the high inflammation in T2DM patients.

## 5. Conclusions

In summary, our findings show that a long-term HFHF diet induces metabolic disorders, oxidation, and inflammation, as well as changes in islet size and irregular secretory functions in the pancreas. ScRNA-seq analysis of pancreatic cells revealed that the long-term HFHF diet resulted in decreased islet cell counts and the enrichment of hormone secretion biological processes, as well as of the oxidative and inflammatory pathways in islet alpha, beta, and PP cells. During the aging process, the expression of major hormone-synthesis genes is upregulated, and the transcriptional level of most oxidoreductases is decreased in islet cells. This study decoded the long-term HFHF diet and aging-associated structural and transcriptomic changes that underlie pancreatic islet functional decay at a single-cell resolution and indicated that HFHF diet-induced inflammation occurred after the accumulation of oxidative stress, suggesting an avenue to prevent improper diet-related diabetes.

## Data Availability

The data discussed in this publication were deposited in NCBI’s Gene Expression Omnibus and are accessible through GEO Series accession number GSE201900 (https://www.ncbi.nlm.nih.gov/geo/query/acc.cgi?acc=GSE201900 (accessed on 19 May 2022)).

## Supplementary Materials

The following supporting information can be downloaded at https://www.mdpi.com/article/10.3390/nu14112181/s1. Figure S1: Evolution of weight in this study; Figure S2: Evolution of hepatic complications in study; Figure S3: Level of pancreas-related endocrine proteins during the intraperitoneal glucose tolerance test (IpGTT); Figure S4: Morphometric analysis of pancreatic islet size and density in this study; Figure S5: Adipocyte infiltration in the pancreas in this study. Figure S6: Basic information from scRNA-Seq Seurat Analysis of diabetic rats. Figure S7: Distinct cell populations with transcriptional signatures in pancreatic cells determined by scRNA-Seq analysis of diabetic rats. Figure S8: Gene Ontology (GO) terms of secretory cells; Figure S9: Distinct cell populations with transcriptional signatures in secretory cells determined by a scRNA-Seq analysis of diabetic rats. Table S1: Ingredients of the diet (%). Table S2: Evolution of physiological, metabolic, oxidative, and inflammatory parameters during the study.

## References

[1] Global Report on Diabetes. https://www.who.int/publications/i/item/who-nmh-nvi-16.3.

[2] American Diabetes Association (2013). Diagnosis and classification of diabetes mellitus. Diabetes Care.

[3] Saeedi P., Salpea P., Karuranga S., Petersohn I., Malanda B., Gregg E.W., Unwin N., Wild S.H., Williams R. (2020). Mortality attributable to diabetes in 20–79 years old adults, 2019 estimates: Results from the International Diabetes Federation Diabetes Atlas. Diabetes Res. Clin. Pract..

[4] Ley S.H., Hamdy O., Mohan V., Hu F.B. (2014). Prevention and management of type 2 diabetes: Dietary components and nutritional strategies. Lancet.

[5] Welsh J.A., Sharma A., Abramson J.L., Vaccarino V., Gillespie C., Vos M.B. (2010). Caloric sweetener consumption and dyslipidemia among US adults. JAMA.

[6] Softic S., Stanhope K.L., Boucher J., Divanovic S., Lanaspa M.A., Johnson R.J., Kahn C.R. (2020). Fructose and hepatic insulin resistance. Crit. Rev. Clin. Lab. Sci..

[7] Tappy L., Le K.A. (2010). Metabolic effects of fructose and the worldwide increase in obesity. Physiol. Rev..

[8] Ludwig D.S. (2000). Dietary glycemic index and obesity. J. Nutr..

[9] Dekker M.J., Su Q., Baker C., Rutledge A.C., Adeli K. (2010). Fructose: A highly lipogenic nutrient implicated in insulin resistance, hepatic steatosis, and the metabolic syndrome. Am. J. Physiol. Endocrinol. Metab..

[10] Sun X., Han F., Lu Q., Li X., Ren D., Zhang J., Han Y., Xiang Y.K., Li J. (2020). Empagliflozin Ameliorates Obesity-Related Cardiac Dysfunction by Regulating Sestrin2-Mediated AMPK-mTOR Signaling and Redox Homeostasis in High-Fat Diet-Induced Obese Mice. Diabetes.

[11] DiNicolantonio J.J., Lucan S.C., O’Keefe J.H. (2016). The Evidence for Saturated Fat and for Sugar Related to Coronary Heart Disease. Prog. Cardiovasc. Dis..

[12] Arisawa K., Uemura H., Yamaguchi M., Nakamoto M., Hiyoshi M., Sawachika F., Katsuura-Kamano S. (2014). Associations of dietary patterns with metabolic syndrome and insulin resistance: A cross-sectional study in a Japanese population. J. Med. Investig..

[13] van Dam R.M., Grievink L., Ocke M.C., Feskens E.J. (2003). Patterns of food consumption and risk factors for cardiovascular disease in the general Dutch population. Am. J. Clin. Nutr..

[14] Darani Zad N., Mohd Yusof R., Esmaili H., Jamaluddin R., Mohseni F. (2015). Association of dietary pattern with biochemical blood profiles and bodyweight among adults with Type 2 diabetes mellitus in Tehran, Iran. J. Diabetes Metab. Disord..

[15] Esmaillzadeh A., Kimiagar M., Mehrabi Y., Azadbakht L., Hu F.B., Willett W.C. (2007). Dietary patterns, insulin resistance, and prevalence of the metabolic syndrome in women. Am. J. Clin. Nutr..

[16] van Dam R.M., Rimm E.B., Willett W.C., Stampfer M.J., Hu F.B. (2002). Dietary patterns and risk for type 2 diabetes mellitus in U.S. men. Ann. Intern. Med..

[17] Lutsey P.L., Steffen L.M., Stevens J. (2008). Dietary intake and the development of the metabolic syndrome: The Atherosclerosis Risk in Communities study. Circulation.

[18] Schulze M.B., Hoffmann K., Manson J.E., Willett W.C., Meigs J.B., Weikert C., Heidemann C., Colditz G.A., Hu F.B. (2005). Dietary pattern, inflammation, and incidence of type 2 diabetes in women. Am. J. Clin. Nutr..

[19] Wirfalt E., Hedblad B., Gullberg B., Mattisson I., Andren C., Rosander U., Janzon L., Berglund G. (2001). Food patterns and components of the metabolic syndrome in men and women: A cross-sectional study within the Malmo Diet and Cancer cohort. Am. J. Epidemiol..

[20] Medina-Remon A., Kirwan R., Lamuela-Raventos R.M., Estruch R. (2018). Dietary patterns and the risk of obesity, type 2 diabetes mellitus, cardiovascular diseases, asthma, and neurodegenerative diseases. Crit. Rev. Food Sci. Nutr..

[21] Imamura F., O’Connor L., Ye Z., Mursu J., Hayashino Y., Bhupathiraju S.N., Forouhi N.G. (2015). Consumption of sugar sweetened beverages, artificially sweetened beverages, and fruit juice and incidence of type 2 diabetes: Systematic review, meta-analysis, and estimation of population attributable fraction. BMJ.

[22] Malik V.S., Popkin B.M., Bray G.A., Despres J.P., Willett W.C., Hu F.B. (2010). Sugar-sweetened beverages and risk of metabolic syndrome and type 2 diabetes: A meta-analysis. Diabetes Care.

[23] InterAct C., Romaguera D., Norat T., Wark P.A., Vergnaud A.C., Schulze M.B., van Woudenbergh G.J., Drogan D., Amiano P., Molina-Montes E. (2013). Consumption of sweet beverages and type 2 diabetes incidence in European adults: Results from EPIC-InterAct. Diabetologia.

[24] Brownlee M. (2001). Biochemistry and molecular cell biology of diabetic complications. Nature.

[25] Betteridge D.J. (2000). What is oxidative stress?. Metabolism.

[26] Gawlik K., Naskalski J.W., Fedak D., Pawlica-Gosiewska D., Grudzien U., Dumnicka P., Malecki M.T., Solnica B. (2016). Markers of Antioxidant Defense in Patients with Type 2 Diabetes. Oxidative Med. Cell. Longev..

[27] Bandeira Sde M., Guedes Gda S., da Fonseca L.J., Pires A.S., Gelain D.P., Moreira J.C., Rabelo L.A., Vasconcelos S.M., Goulart M.O. (2012). Characterization of blood oxidative stress in type 2 diabetes mellitus patients: Increase in lipid peroxidation and SOD activity. Oxidative Med. Cell. Longev..

[28] Broedbaek K., Siersma V., Henriksen T., Weimann A., Petersen M., Andersen J.T., Jimenez-Solem E., Stovgaard E.S., Hansen L.J., Henriksen J.E. (2011). Urinary markers of nucleic acid oxidation and long-term mortality of newly diagnosed type 2 diabetic patients. Diabetes Care.

[29] Broedbaek K., Siersma V., Henriksen T., Weimann A., Petersen M., Andersen J.T., Jimenez-Solem E., Hansen L.J., Henriksen J.E., Bonnema S.J. (2013). Association between urinary markers of nucleic acid oxidation and mortality in type 2 diabetes: A population-based cohort study. Diabetes Care.

[30] Wang W.X., Luo S.B., Xia M.M., Mao Y.H., Zhou X.Y., Jiang P., Jiang H.Y., Dai D.P., Li C.B., Hu G.X. (2015). Analysis of the oxidative damage of DNA, RNA, and their metabolites induced by hyperglycemia and related nephropathy in Sprague Dawley rats. Free Radic. Res..

[31] Al-Awar A., Kupai K., Veszelka M., Szucs G., Attieh Z., Murlasits Z., Torok S., Posa A., Varga C. (2016). Experimental Diabetes Mellitus in Different Animal Models. J. Diabetes Res..

[32] Lenzen S. (2008). The mechanisms of alloxan- and streptozotocin-induced diabetes. Diabetologia.

[33] Lozano I., Van der Werf R., Bietiger W., Seyfritz E., Peronet C., Pinget M., Jeandidier N., Maillard E., Marchioni E., Sigrist S. (2016). High-fructose and high-fat diet-induced disorders in rats: Impact on diabetes risk, hepatic and vascular complications. Nutr. Metab..

[34] Dal S., Van der Werf R., Walter C., Bietiger W., Seyfritz E., Mura C., Peronet C., Legrandois J., Werner D., Ennahar S. (2018). Treatment of NASH with Antioxidant Therapy: Beneficial Effect of Red Cabbage on Type 2 Diabetic Rats. Oxidative Med. Cell. Longev..

[35] Van der Werf R., Walter C., Bietiger W., Seyfritz E., Mura C., Peronet C., Legrandois J., Werner D., Ennahar S., Digel F. (2018). Beneficial effects of cherry consumption as a dietary intervention for metabolic, hepatic and vascular complications in type 2 diabetic rats. Cardiovasc. Diabetol..

[36] Liao H., Chou L.M., Chien Y.W., Wu C.H., Chang J.S., Lin C.I., Lin S.H. (2017). Grape powder consumption affects the expression of neurodegeneration-related brain proteins in rats chronically fed a high-fructose-high-fat diet. J. Nutr. Biochem..

[37] Chou L.M., Lin C.I., Chen Y.H., Liao H., Lin S.H. (2016). A diet containing grape powder ameliorates the cognitive decline in aged rats with a long-term high-fructose-high-fat dietary pattern. J. Nutr. Biochem..

[38] Afifi N.A., Ramadan A., Erian E.Y., Saleh D.O., Sedik A.A., Badawi M., El Hotaby W. (2017). Trigonelline attenuates hepatic complications and molecular alterations in high-fat high-fructose diet-induced insulin resistance in rats. Can. J. Physiol. Pharmacol..

[39] Casagrande B.P., Gomes M.F.P., Moura E.O.C., Santos A.C.C., Kubota M.C., Ribeiro D.A., Pisani L.P., Medeiros A., Estadella D. (2019). Age-dependent hepatic alterations induced by a high-fat high-fructose diet. Inflamm. Res..

[40] Vidal E., Lalarme E., Maire M.A., Febvret V., Gregoire S., Gambert S., Acar N., Bretillon L. (2019). Early impairments in the retina of rats fed with high fructose/high fat diet are associated with glucose metabolism deregulation but not dyslipidaemia. Sci. Rep..

[41] Liu M., Weiss M.A., Arunagiri A., Yong J., Rege N., Sun J., Haataja L., Kaufman R.J., Arvan P. (2018). Biosynthesis, structure, and folding of the insulin precursor protein. Diabetes Obes. Metab..

[42] Rains J.L., Jain S.K. (2011). Oxidative stress, insulin signaling, and diabetes. Free Radic. Biol. Med..

[43] Chansela P., Potip B., Weerachayaphorn J., Kangwanrangsan N., Chukijrungroat N., Saengsirisuwan V. (2022). Morphological alteration of the pancreatic islet in ovariectomized rats fed a high-fat high-fructose diet. Histochem. Cell Biol..

[44] O’Dowd J.F. (2009). The isolation and purification of rodent pancreatic islets of Langerhans. Methods Mol. Biol..

[45] Li D.S., Yuan Y.H., Tu H.J., Liang Q.L., Dai L.J. (2009). A protocol for islet isolation from mouse pancreas. Nat. Protoc..

[46] Chang N., Tian L., Ji X., Zhou X., Hou L., Zhao X., Yang Y., Yang L., Li L. (2019). Single-Cell Transcriptomes Reveal Characteristic Features of Mouse Hepatocytes with Liver Cholestatic Injury. Cells.

[47] Zhao T., Fu Y., Zhu J., Liu Y., Zhang Q., Yi Z., Chen S., Jiao Z., Xu X., Xu J. (2018). Single-Cell RNA-Seq Reveals Dynamic Early Embryonic-like Programs during Chemical Reprogramming. Cell Stem Cell.

[48] Leibiger I.B., Leibiger B., Berggren P.O. (2008). Insulin signaling in the pancreatic beta-cell. Annu. Rev. Nutr..

[49] van der Kooi J.B., van Wanroy P.J., De Jonge M.C., Kornelis J.A. (1984). Time separation between cough pulses in bladder, rectum and urethra in women. J. Urol..

[50] Sims E.K., Carr A.L.J., Oram R.A., DiMeglio L.A., Evans-Molina C. (2021). 100 years of insulin: Celebrating the past, present and future of diabetes therapy. Nat. Med..

[51] Madsen O.D., Frank B.H., Steiner D.F. (1984). Human proinsulin-specific antigenic determinants identified by monoclonal antibodies. Diabetes.

[52] Gray I.P., Siddle K., Frank B.H., Hales C.N. (1987). Characterization and use in immunoradiometric assay of monoclonal antibodies directed against human proinsulin. Diabetes.

[53] Imai S., Takahashi T., Naito S., Yamauchi A., Okada C., Notsu Y., Sakikawa I., Hatanaka M., Iwasaki T., Morita A. (2015). Development of a novel immunoassay specific for mouse intact proinsulin. Anal. Biochem..

[54] Ozawa S., Katsuta H., Suzuki K., Takahashi K., Tanaka T., Sumitani Y., Nishida S., Yoshimoto K., Ishida H. (2014). Estimated proinsulin processing activity of prohormone convertase (PC) 1/3 rather than PC2 is decreased in pancreatic beta-cells of type 2 diabetic patients. Endocr. J..

[55] Martens G.A., Jiang L., Hellemans K.H., Stange G., Heimberg H., Nielsen F.C., Sand O., Van Helden J., Van Lommel L., Schuit F. (2011). Clusters of conserved beta cell marker genes for assessment of beta cell phenotype. PLoS ONE.

[56] Zhu Y., Liu Q., Zhou Z., Ikeda Y. (2017). PDX1, Neurogenin-3, and MAFA: Critical transcription regulators for beta cell development and regeneration. Stem Cell Res. Ther..

[57] Kotnik P., Fischer-Posovszky P., Wabitsch M. (2011). RBP4: A controversial adipokine. Eur. J. Endocrinol..

[58] Yeo G.S.H., Chao D.H.M., Siegert A.M., Koerperich Z.M., Ericson M.D., Simonds S.E., Larson C.M., Luquet S., Clarke I., Sharma S. (2021). The melanocortin pathway and energy homeostasis: From discovery to obesity therapy. Mol. Metab..

[59] Jackson R.S., Creemers J.W., Ohagi S., Raffin-Sanson M.L., Sanders L., Montague C.T., Hutton J.C., O’Rahilly S. (1997). Obesity and impaired prohormone processing associated with mutations in the human prohormone convertase 1 gene. Nat. Genet..

[60] Shum M., Pinard S., Guimond M.O., Labbe S.M., Roberge C., Baillargeon J.P., Langlois M.F., Alterman M., Wallinder C., Hallberg A. (2013). Angiotensin II type 2 receptor promotes adipocyte differentiation and restores adipocyte size in high-fat/high-fructose diet-induced insulin resistance in rats. Am. J. Physiol. Endocrinol. Metab..

[61] Odermatt A. (2011). The Western-style diet: A major risk factor for impaired kidney function and chronic kidney disease. Am. J. Physiol. Ren. Physiol..

[62] Hull R.L., Kodama K., Utzschneider K.M., Carr D.B., Prigeon R.L., Kahn S.E. (2005). Dietary-fat-induced obesity in mice results in beta cell hyperplasia but not increased insulin release: Evidence for specificity of impaired beta cell adaptation. Diabetologia.

[63] Linnemann A.K., Baan M., Davis D.B. (2014). Pancreatic beta-cell proliferation in obesity. Adv. Nutr..

[64] Medina-Gomez G., Gray S.L., Yetukuri L., Shimomura K., Virtue S., Campbell M., Curtis R.K., Jimenez-Linan M., Blount M., Yeo G.S. (2007). PPAR gamma 2 prevents lipotoxicity by controlling adipose tissue expandability and peripheral lipid metabolism. PLoS Genet..

[65] Rosen E.D., Kulkarni R.N., Sarraf P., Ozcan U., Okada T., Hsu C.H., Eisenman D., Magnuson M.A., Gonzalez F.J., Kahn C.R. (2003). Targeted elimination of peroxisome proliferator-activated receptor gamma in beta cells leads to abnormalities in islet mass without compromising glucose homeostasis. Mol. Cell Biol..

[66] Vidal-Puig A., Jimenez-Linan M., Lowell B.B., Hamann A., Hu E., Spiegelman B., Flier J.S., Moller D.E. (1996). Regulation of PPAR gamma gene expression by nutrition and obesity in rodents. J. Clin. Investig..

[67] He X., Memczak S., Qu J., Belmonte J.C.I., Liu G.H. (2020). Single-cell omics in ageing: A young and growing field. Nat. Metab..

[68] Wang Y.J., Schug J., Won K.J., Liu C., Naji A., Avrahami D., Golson M.L., Kaestner K.H. (2016). Single-Cell Transcriptomics of the Human Endocrine Pancreas. Diabetes.

[69] Enge M., Arda H.E., Mignardi M., Beausang J., Bottino R., Kim S.K., Quake S.R. (2017). Single-Cell Analysis of Human Pancreas Reveals Transcriptional Signatures of Aging and Somatic Mutation Patterns. Cell.

[70] Xin Y., Kim J., Okamoto H., Ni M., Wei Y., Adler C., Murphy A.J., Yancopoulos G.D., Lin C., Gromada J. (2016). RNA Sequencing of Single Human Islet Cells Reveals Type 2 Diabetes Genes. Cell Metab..

[71] Shoelson S.E., Lee J., Goldfine A.B. (2006). Inflammation and insulin resistance. J. Clin. Investig..

[72] Lontchi-Yimagou E., Sobngwi E., Matsha T.E., Kengne A.P. (2013). Diabetes mellitus and inflammation. Curr. Diabetes Rep..

[73] Donath M.Y., Schumann D.M., Faulenbach M., Ellingsgaard H., Perren A., Ehses J.A. (2008). Islet inflammation in type 2 diabetes: From metabolic stress to therapy. Diabetes Care.

