# Supplementary material for: Long-Term High-Fat High-Fructose Diet Induces Type 2 Diabetes in Rats through Oxidative Stress
Source: Nutrients. 2022 May 24;14(11):2181. doi: 10.3390/nu14112181 (PMC9182436; doi:10.3390/nu14112181)
Supplement: Supplementary file 1 [file nutrients-14-02181-s001.zip › Tables/Table S1.pdf]

Table S1. Ingredients of the diet (%).

	Normal diet%(w/w) *	HFHF diet%(w/w) *
Fructose (in water)	0.00	20.00
Wheat flour	33.10	26.08
Corn	40.84	32.18
Fish meal	2.00	1.58
Soybean	16.06	12.66
Soybean oil	2.00	1.58
Cholesterol	0.00	1.00
Lard	0.00	10.00
Egg yolk powder	0.00	10.00
Sodium cholate	0.00	0.20
Total ash	6.00	4.73

* The diets were formulated based on GB14924 and D12079B diet.
HFHF, high-fat, high-fructose diet.
