# Supplementary material for: Long-Term High-Fat High-Fructose Diet Induces Type 2 Diabetes in Rats through Oxidative Stress
Source: Nutrients. 2022 May 24;14(11):2181. doi: 10.3390/nu14112181 (PMC9182436; doi:10.3390/nu14112181)
Supplement: Supplementary file 1 [file nutrients-14-02181-s001.zip › Tables/Table S2.pdf]

**Table S2. Evolution of physiological, metabolic, oxidative and inflammatory parameters during the study.**

| | Baseline | Month 3 | | Month 18 | |
|---|---|---|---|---|---|
| | | Control | HFHF | Control | HFHF |
| **Physiological variables** | | | | | |
| Weight (g) | 280.75 ± 8.19 | 545.13 ± 15.05 | 616.25 ± 14.18**** | 831.63 ± 75.03 | 1188.63 ± 70.50**** |
| Body mass index (BMI; g/cm$^2$) | 5.03 ± 0.18 | 7.48 ± 0.31 | 8.78 ± 0.29**** | 8.68 ± 0.89 | 11.21 ± 0.51**** |
| Abdominal circumference (cm) | 13.48 ± 0.61 | 17.68 ± 1.71 | 23.56 ± 1.68**** | 28.30 ± 2.35 | 34.26 ± 2.14**** |
| Liver weight (g) | 8.00 ± 1.42 | 16.53 ± 1.36 | 23.89 ± 2.53**** | 23.33 ± 1.88 | 52.75 ± 7.59**** |
| Relative liver weight (%) | 2.84 ± 0.44 | 3.03 ± 0.19 | 3.87 ± 0.33**** | 2.82 ± 0.28 | 4.45 ± 0.72**** |
| **Plasmatic metabolic parameters** | | | | | |
| GSP (mmol/L) | 1.26 ± 0.11 | 1.62 ± 0.23 | 2.60 ± 0.56*** | 1.54 ± 0.16 | 4.03 ± 0.80**** |
| Glucose (mmol/L) | 10.72 ± 2.08 | 10.34 ± 1.48 | 14.95 ± 3.02** | 14.42 ± 1.57 | 20.63 ± 3.60*** |
| TG (mmol/L) | 0.74 ± 0.18 | 1.31 ± 0.38 | 1.70 ± 0.58 | 1.02 ± 0.17 | 3.00 ± 1.04*** |
| T-CHO (mmol/L) | 1.96 ± 0.19 | 1.99 ± 0.42 | 3.30 ± 1.02** | 3.36 ± 0.85 | 8.39 ± 2.92*** |
| LDL-C (mmol/L) | 0.87 ± 0.15 | 0.73 ± 0.16 | 1.00 ± 0.28* | 0.99 ± 0.31 | 3.03 ± 1.70** |
| Pyruvic acid (µmol/mL) | 0.39 ± 0.07 | 0.44 ± 0.08 | 0.92 ± 0.24*** | 0.43 ± 0.04 | 1.02 ± 0.20**** |
| **Plasmatic oxidative parameters** | | | | | |
| OX-LDL (µg/mL) | 8.94 ± 0.94 | 11.24 ± 2.25 | 11.81 ± 2.33 | 12.93 ± 2.51 | 16.69 ± 1.33** |
| LPO (µmol/L) | 5.52 ± 0.42 | 6.32 ± 1.00 | 4.99 ± 0.94* | 7.15 ± 1.85 | 10.19 ± 3.04* |
| SOD (U/mL) | 91.44 ± 6.47 | 84.39 ± 8.54 | 123.31 ± 10.40**** | 102.39 ± 13.02 | 135.01 ± 10.09**** |
| LDH (U/L) | 6416.78 ± 1725.93 | 8037.58 ± 1457.78 | 11422.82 ± 2656.13** | 9050.34 ± 1475.18 | 13109.40 ± 2692.66** |
| XOD (U/L) | 38.44 ± 3.89 | 42.82 ± 5.64 | 56.90 ± 8.10*** | 39.57 ± 3.34 | 82.04 ± 14.89**** |
| GR (U/L) | 5.02 ± 1.34 | 5.02 ± 1.34 | 6.63 ± 1.59* | 6.43 ± 2.27 | 11.46 ± 3.60** |
| T-AOC (mmol/L) | 0.50 ± 0.23 | 0.68 ± 0.09 | 0.91 ± 0.11*** | 0.55 ± 0.08 | 1.08 ± 0.18**** |

| | | | | | |
|---|---|---|---|---|---|
| $H_2O_2$ (mmol/L) | 102.14 ± 17.26 | 176.34 ± 36.70 | 204.36 ± 38.60 | 145.10 ± 8.70 | 297.04 ± 55.30**** |
| GSH (μmol/L) | 7.88 ± 2.82 | 6.40 ± 1.08 | 5.90 ± 1.57 | 7.06 ± 1.53 | 5.12 ± 2.06* |
| **Inflammatory parameters** | | | | | |
| IL-1β (ng/L) | 15.31 ± 2.00 | 18.12 ± 4.41 | 21.21 ± 6.17 | 20.30 ± 4.62 | 29.01 ± 4.48** |
| IL-6 (ng/L) | 38.56 ± 3.37 | 44.40 ± 7.39 | 54.42 ± 10.21* | 54.92 ± 8.85 | 75.00 ± 5.42**** |
| MCP-1 (ng/mL) | 104.03 ± 15.65 | 118.98 ± 25.54 | 118.39 ± 18.97 | 117.91 ± 14.80 | 169.71 ± 13.39**** |
| TNF-α (ng/L) | 66.40 ± 6.14 | 97.51 ± 17.39 | 99.58 ± 23.04 | 99.46 ± 15.66 | 117.57 ± 12.17* |
| VEGF (ng/L) | 154.56 ± 12.03 | 216.31 ± 32.83 | 247.01 ± 39.68 | 229.51 ± 32.15 | 290.42 ± 24.94*** |
| ICAM1 (ng/mL) | 40.91 ± 8.81 | 58.07 ± 12.72 | 61.43 ± 13.15 | 57.95 ± 14.01 | 75.35 ± 12.45* |

Effects of diet on physiological, metabolic, oxidative and inflammatory parameters during the study at the beginning (Baseline) and after 3 (Month 3) and 18 months (Month 18) of normal (control) and high-fat, high-fructose (HFHF) diets. Relative liver weight, the percentage of liver weight in total body weight; GSP, glycated serum protein; TG, total triglycerides; T-CHO, total cholesterol; LDL-C, low-density lipoprotein cholesterol; OX-LDL, oxidized low density lipoprotein; LPO, lipid hydroperoxide; SOD, superoxide dismutase activity; LDH, lactate dehydrogenase; XOD, xanthine oxidase; GR, glutathione reductase; T-AOC, total antioxidant capacity; GSH, glutathione; $H_2O_2$, hydrogen peroxide; IL-1β, Interleukin-1β; IL-6, Interleukin-6; MCP-1, monocyte chemotactic protein-1; TNF-α, tumor necrosis factor-α; VEGF, vascular endothelial growth factor; ICAM1, intercellular cell adhesion molecule-1. The results are shown as the mean ± SD. *Significant results versus the age-matched control group. *$p<0.05$, **$p<0.01$, ***$p<0.001$, ****$p<0.0001$.
