# Supplementary figures and images for: Long-Term High-Fat High-Fructose Diet Induces Type 2 Diabetes in Rats through Oxidative Stress
Source: Nutrients. 2022 May 24;14(11):2181. doi: 10.3390/nu14112181 (PMC9182436; doi:10.3390/nu14112181)

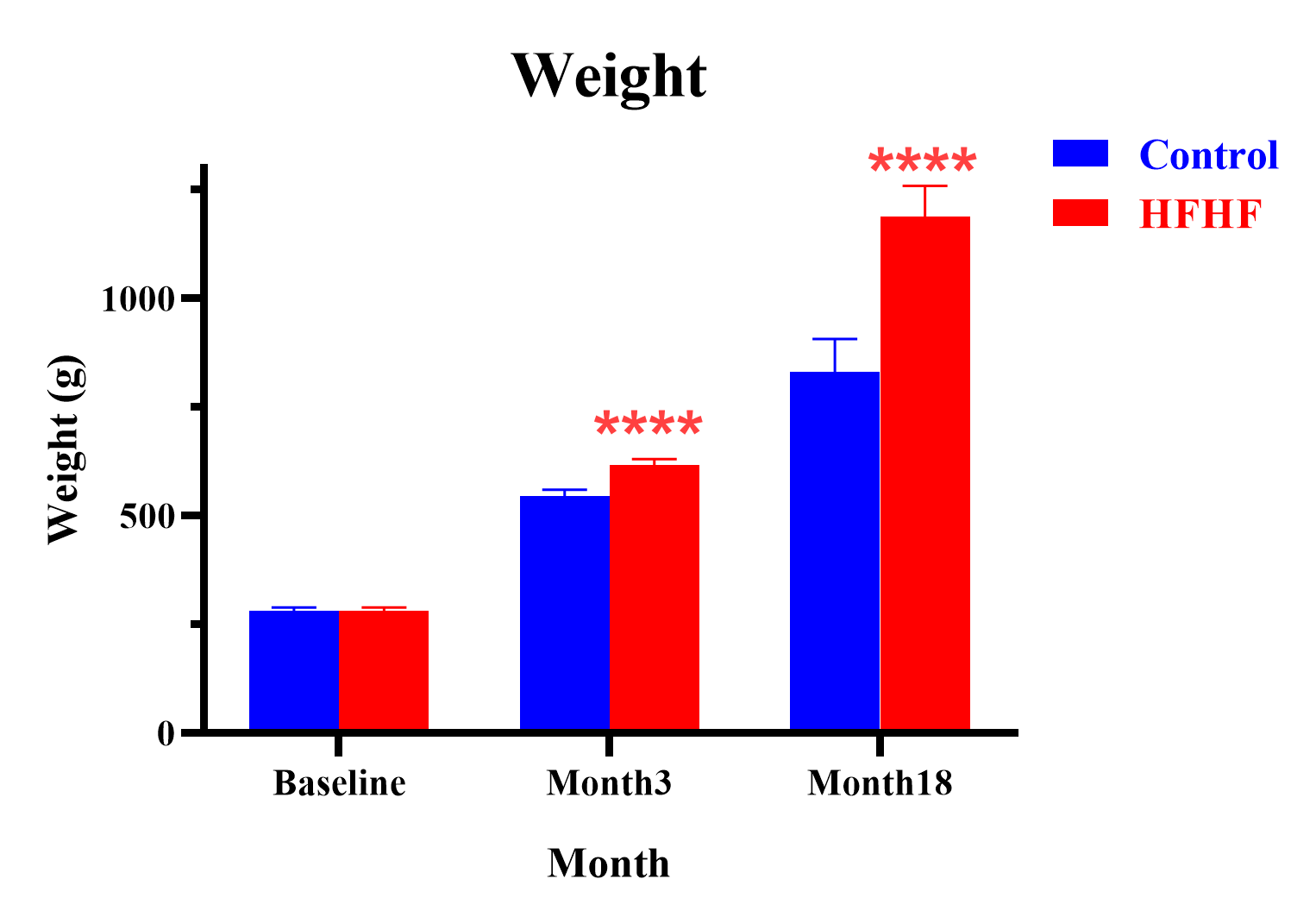

Supplement: Supplementary file 1 [file nutrients-14-02181-s001.zip › Figures/Figure.S1.tif]

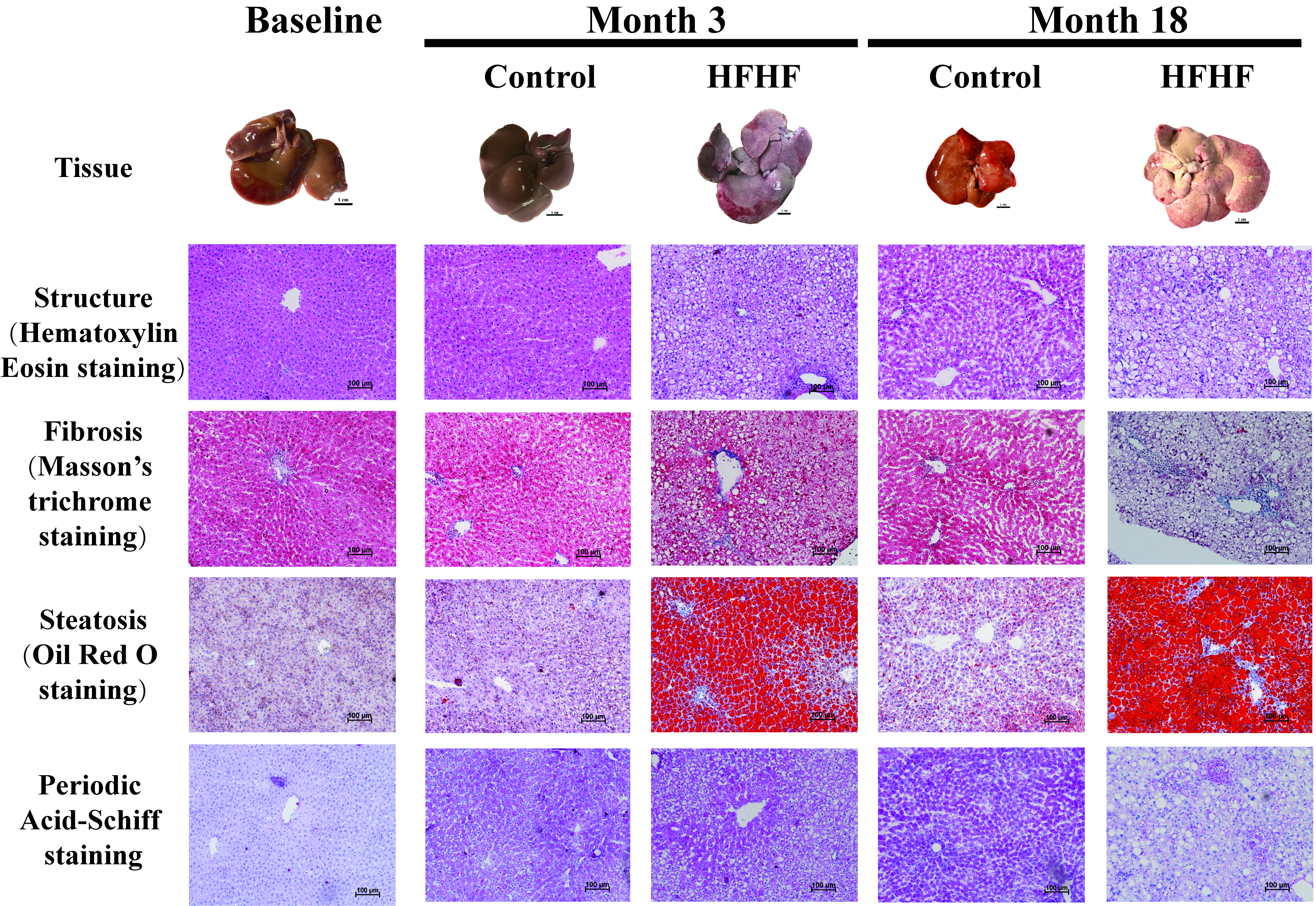

Supplement: Supplementary file 1 [file nutrients-14-02181-s001.zip › Figures/Figure.S2.tif]

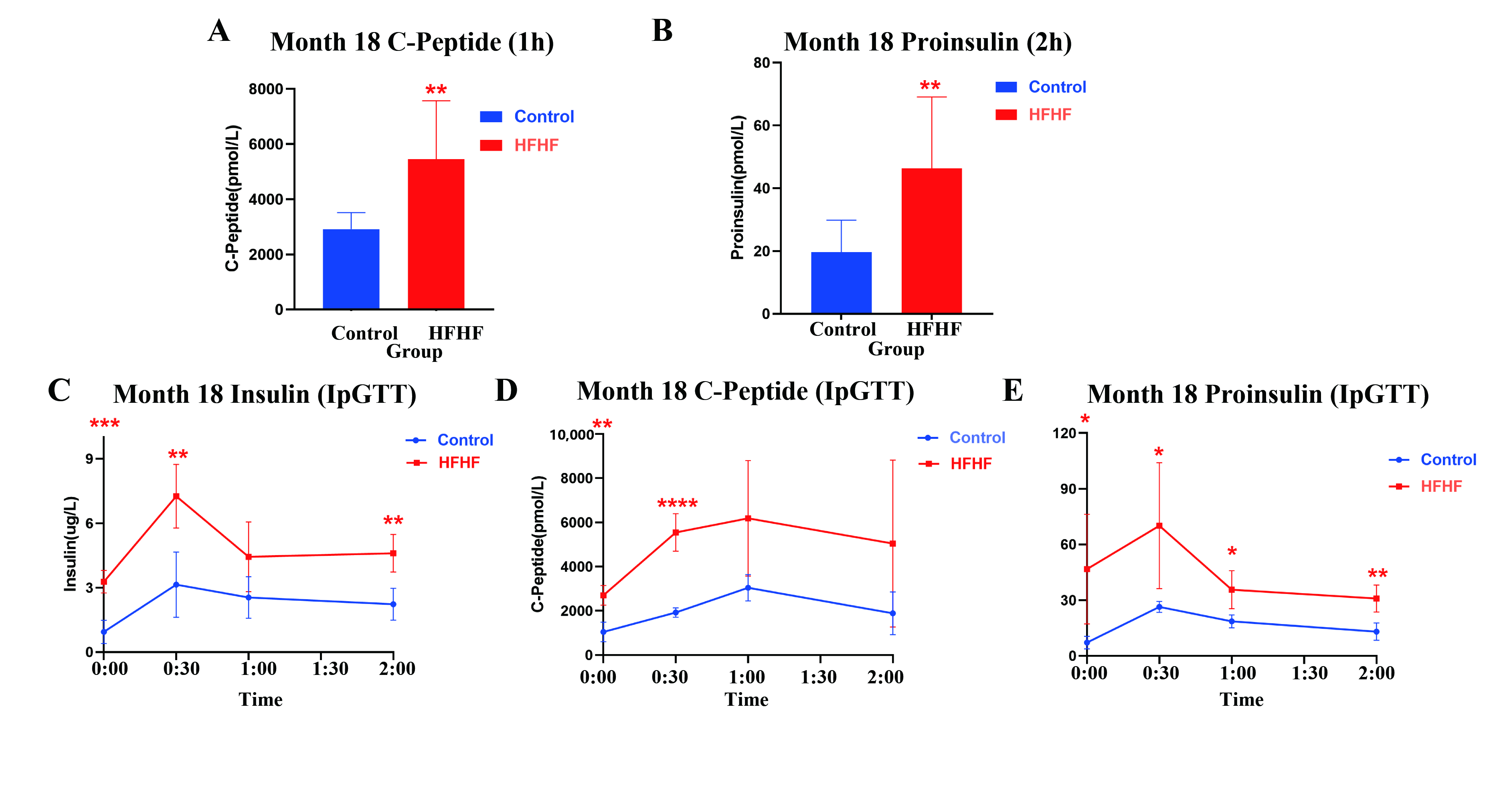

Supplement: Supplementary file 1 [file nutrients-14-02181-s001.zip › Figures/Figure.S3.tif]

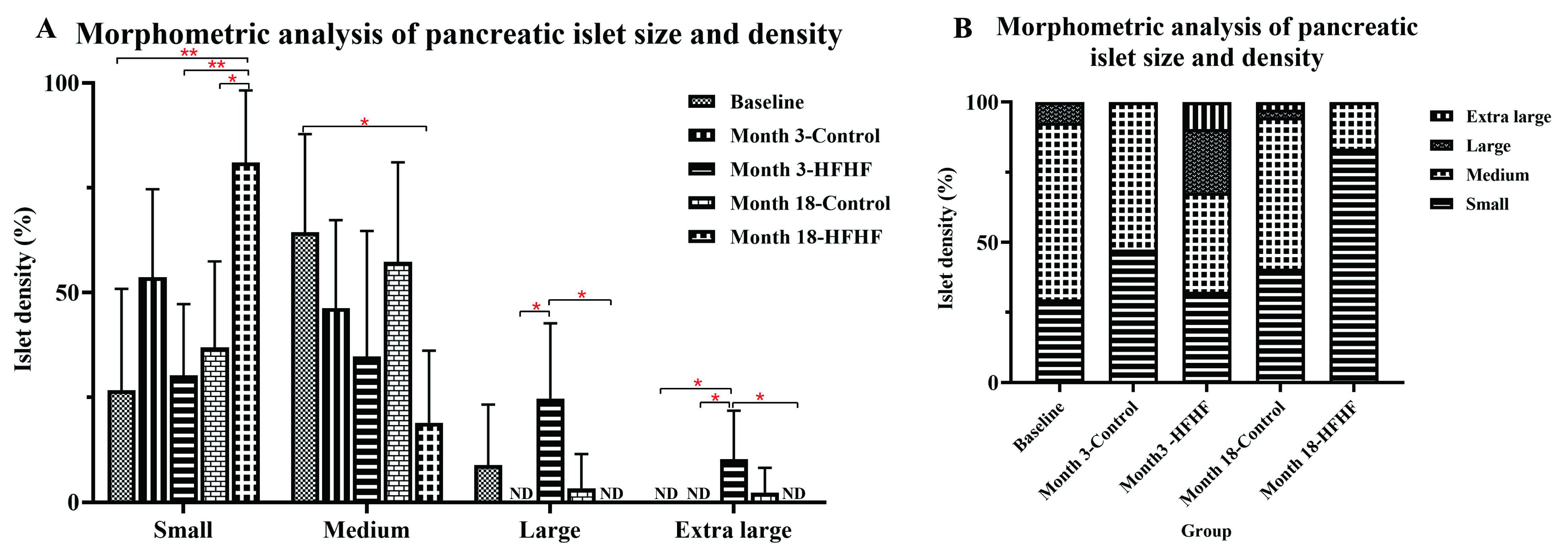

Supplement: Supplementary file 1 [file nutrients-14-02181-s001.zip › Figures/Figure.S4.tif]

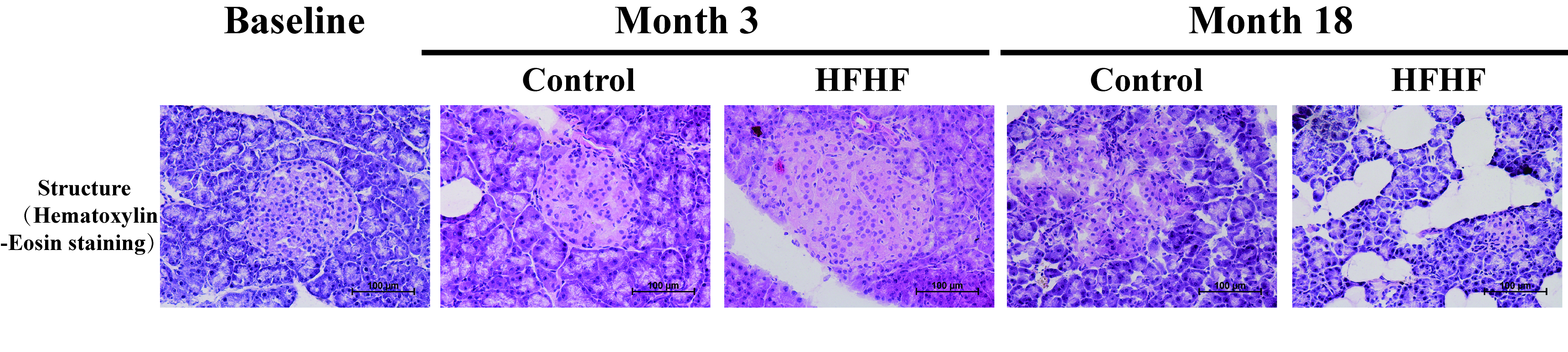

Supplement: Supplementary file 1 [file nutrients-14-02181-s001.zip › Figures/Figure.S5.tif]

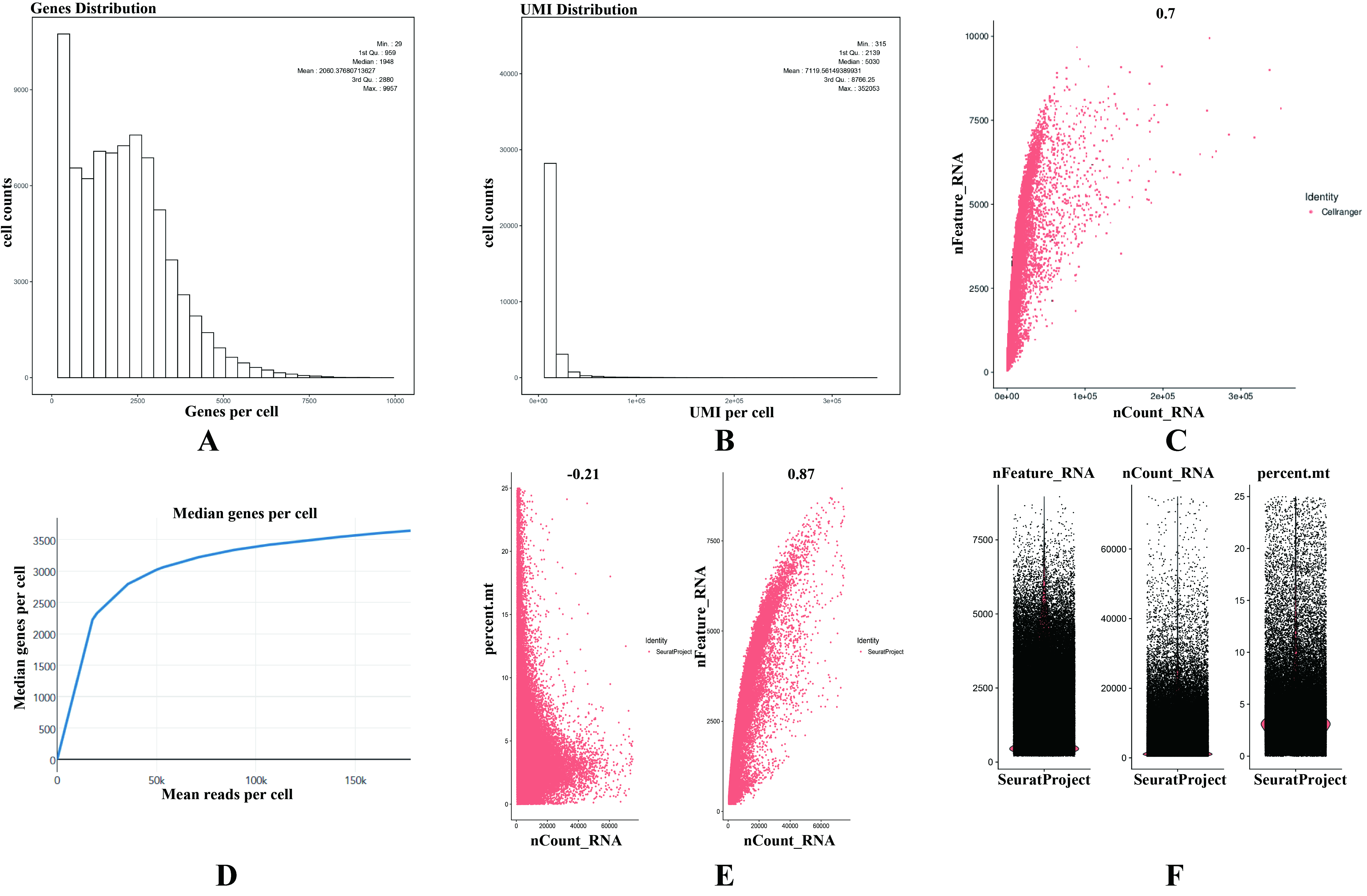

Supplement: Supplementary file 1 [file nutrients-14-02181-s001.zip › Figures/Figure.S6.tif]

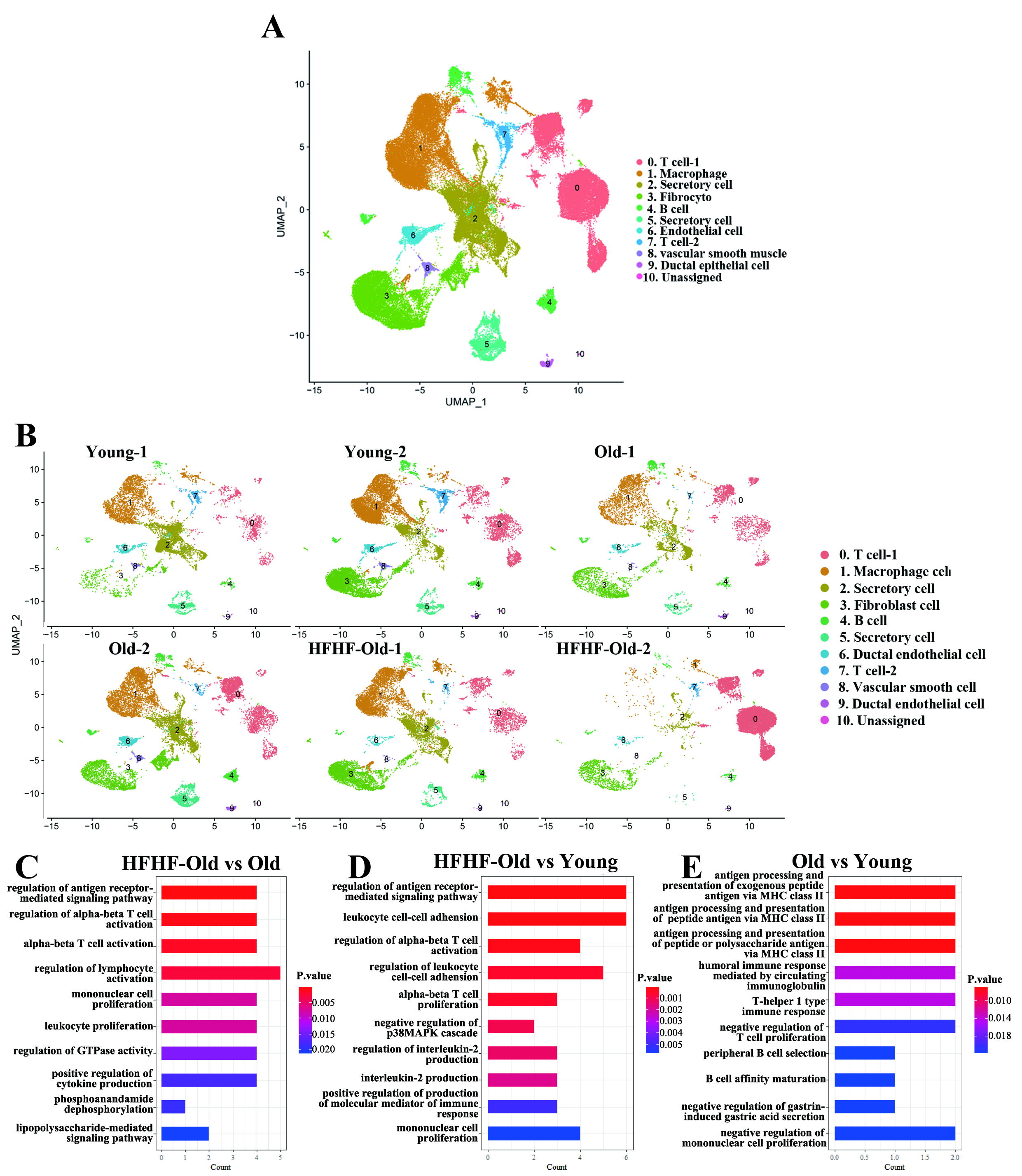

Supplement: Supplementary file 1 [file nutrients-14-02181-s001.zip › Figures/Figure.S7.tif]

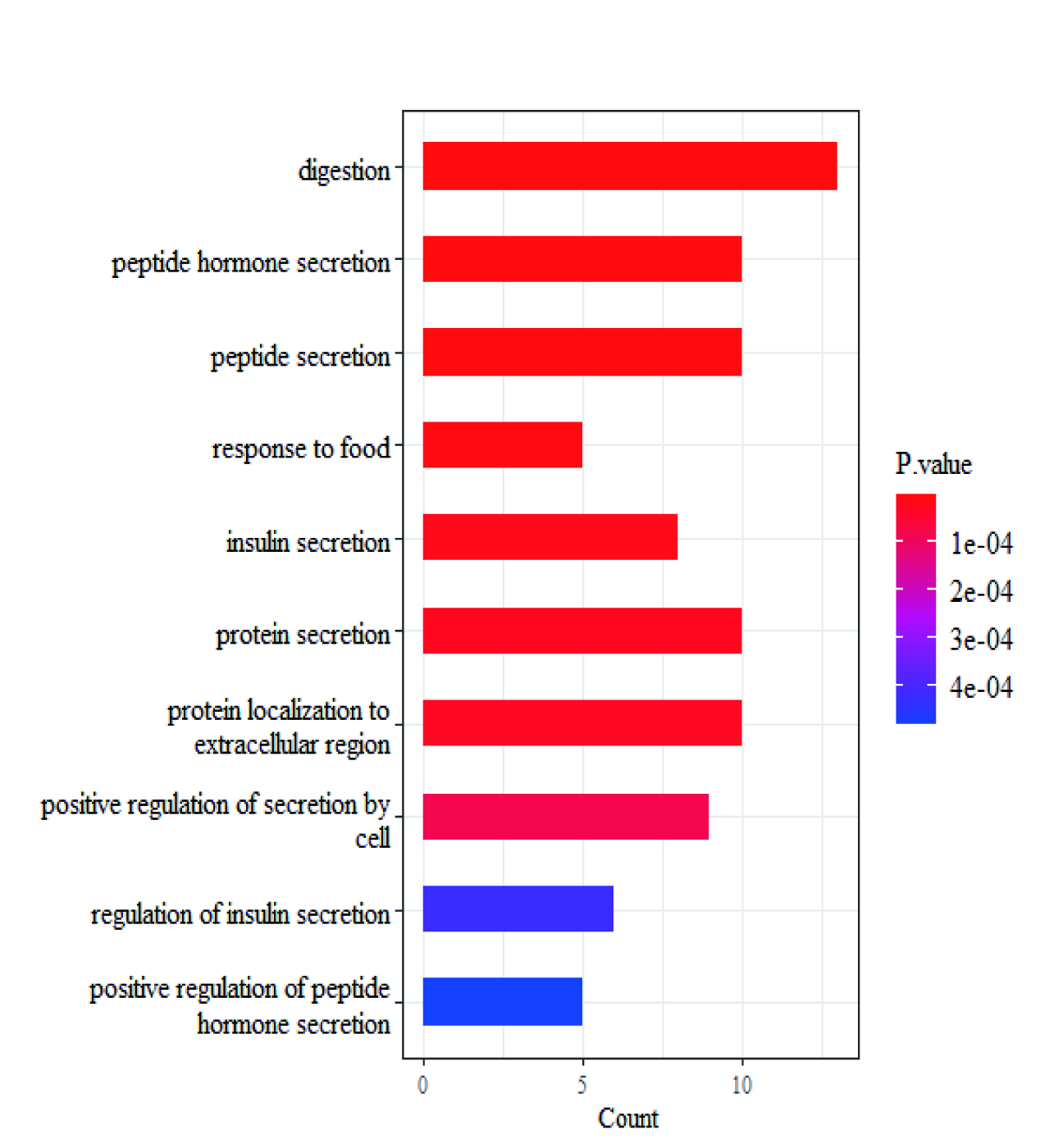

Supplement: Supplementary file 1 [file nutrients-14-02181-s001.zip › Figures/Figure.S8.tif]

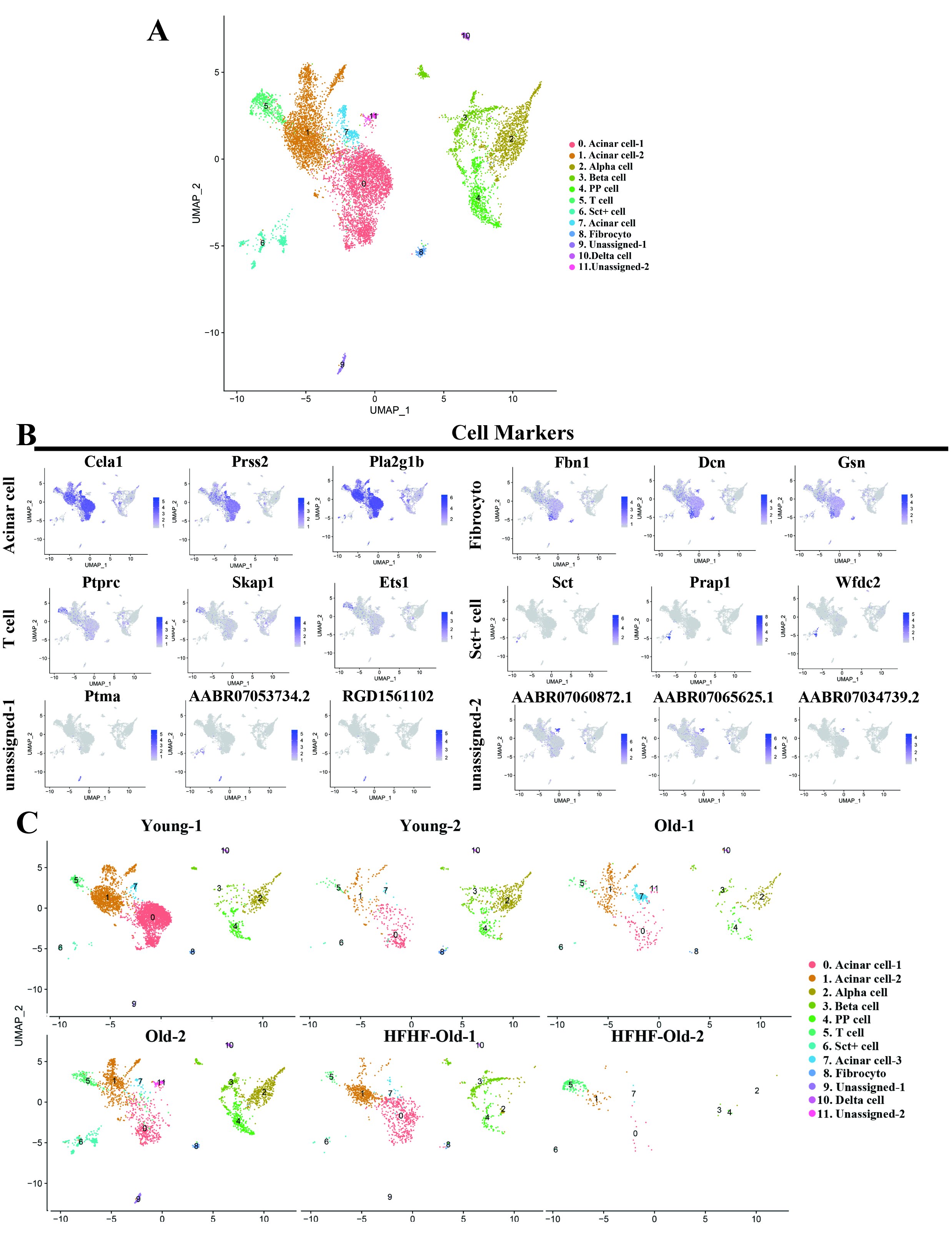

Supplement: Supplementary file 1 [file nutrients-14-02181-s001.zip › Figures/Figure.S9.tif]
